# Systematic screening for atrial fibrillation with non-invasive devices: a systematic review and meta-analysis

**DOI:** 10.1016/j.lanepe.2025.101298

**Published:** 2025-04-11

**Authors:** Ali Wahab, Ramesh Nadarajah, Harriet Larvin, Maryum Farooq, Keerthenan Raveendra, Mohammad Haris, Umbreen Nadeem, Tobin Joseph, Asad Bhatty, Chris Wilkinson, Kamlesh Khunti, Rajesh Vedanthan, A John Camm, Emma Svennberg, Gregory YH. Lip, Ben Freedman, Jianhua Wu, Chris P. Gale

**Affiliations:** a Leeds Institute for Cardiovascular and Metabolic Medicine, University of Leeds, UK; b Leeds Institute of Data Analytics, University of Leeds, UK; c Department of Cardiology, Leeds Teaching Hospitals NHS Trust, Leeds, UK; d Wolfson Institute of Population Health, Queen Mary University of London, UK; e Department of Cardiology, Calderdale and Huddersfield NHS Foundation Trust, Huddersfield, UK; f Faculty of Medicine and Health, University of Leeds, Leeds, UK; g Department of Cardiology, Bradford Teaching Hospitals NHS Foundation Trust, UK; h Department of Cardiology, Mid Yorkshire Teaching NHS Trust, Wakefield, UK; i Department of Cardiology, James Cook Teaching Hospital, South Tees NHS Foundation Trust, UK; j College of Life Sciences, University of Leicester, UK; k Department of Population Health, New York University School of Medicine, New York, USA; l Cardiovascular Clinical Academic Group, St George’s University of London, London, UK; m Department of Medicine, Karolinska Institutet, Karolinska University Hospital, Stockholm, Sweden; n Liverpool Centre for Cardiovascular Science at University of Liverpool, Liverpool John Moores University and Liverpool Heart & Chest Hospital, Liverpool, UK; o Department of Clinical Medicine, Danish Center for Health Services Research, Aalborg University, Aalborg, Denmark; p Sydney Medical School, Charles Perkins Center, and Cardiology Department, Concord Hospital, Heart Research Institute, The University of Sydney, Sydney, Australia

**Keywords:** Atrial fibrillation, Screening, Digital, Public health

## Abstract

**Background:**

Systematic screening individuals with non-invasive devices may improve diagnosis of atrial fibrillation (AF) and reduce adverse clinical events. We systematically reviewed the existing literature to determine the yield of new AF diagnosis associated with systematic AF screening, the relative increase in yield of new AF diagnosis with systematic screening compared to usual care, and the effect of systematic AF screening on clinical outcomes compared with usual care.

**Methods:**

The Medline, Embase, Web of Science and Cochrane Library databases were searched from inception through 1st February 2025 for prospective cohort studies or randomised clinical trials (RCTs) of systematic AF screening with the outcome of incidence of previously undiagnosed AF from screening. Incidence rates (IR) and relative risks were calculated and random effects meta-analysis performed to synthesise rates of AF in prospective cohort studies and RCTs, as well as outcomes in RCTs.

**Findings:**

From 3806 unique records we included 32 studies representing 735,542 participants from 8 RCTs and 24 prospective cohorts. The diagnosis rate for incident AF in prospective cohorts was 2.75% (95% CI 1.87–3.62), and the pooled relative risk in RCTs was 2.22 (95% CI 1.41–3.50). The use of age and NT-proBNP (IR 4.36%, 95% CI 3.77–5.08) or AF risk score classification (4.79%, 95% CI 3.62–6.29) led to higher new AF diagnosis yields than age alone (0.93%, 95% CI 0.28–2.99). Pooled data from RCTs did not demonstrate an effect of screening on death (RR 1.01, 95% CI 0.97–1.05), cardiovascular hospitalisation (1.00, 95% CI 0.97–1.03), stroke (0.95, 95% CI 0.87–1.04) or bleeding (1.08, 95% CI 0.91–1.29).

**Interpretation:**

Systematic screening for AF using non-invasive devices is associated with increased diagnosis of AF, but not reduced adverse clinical events. Screening studies of AF utilising alternative risk stratifications and outcome measures are required.

**Funding:**

10.13039/501100000274British Heart Foundation (grant reference CC/22/250026) and 10.13039/501100000272National Institute for Health and Care Research.

**Research in context**

**Evidence before this study**

We did a preliminary search of Medline, Embase, Web of Science and Cochrane Library for existing reviews evaluating the targeting and conduct of atrial fibrillation (AF) screening trials using the search terms “atrial fibrillation” AND “screening” or their permutations, and limiting the results to reviews and systematic reviews only that were published from database inception to February 2025, without language restrictions. This preliminary investigation revealed only a small number of existing systematic reviews, which did not address the contemporary evidence in the field. Some preceded landmark randomised clinical trials of AF screening, and all preceded the shift in AF screening trials away from an age-only inclusion criteria to also considering other AF or stroke risk factors. Furthermore, some did not provide a quantitative synthesis, which limits an understanding of how conduct and the choice of eligible population may impact on the incidence of previously undiagnosed AF through screening, or outcomes.

**Added value of this study**

This systematic review and meta-analysis of randomised clinical trials and prospective cohort studies of AF screening synthesises data from over 700,000 participants to provide novel insights into how AF screening across European countries may be conducted to optimise yield and recruitment. We demonstrate that the yield of hitherto undiagnosed AF achieved from screening is increased using biomarkers or AF risk assessment. Furthermore, across different geographies and populations, digital approaches to recruitment and conduct of ECG monitoring is shown to be effective at scale. In spite of novel trials published this year, the effect of AF screening on incident stroke is not significant, suggesting that AF screening research may need to consider broader outcomes to fully capture the potential benefit of the intervention.

**Implications of all the available evidence**

The evidence suggests that the use of comprehensive AF risk assessment tools or biomarkers can improve the yield of new AF diagnosed during screening. Improving yield of new AF diagnosed is likely to improve the cost-effectiveness. Multivariable risk scores to define AF risk are available that use data routinely collected in European countries without the requirement for extra appointments or tests, and these may be a suitable approach to make AF screening feasible on both a national and regional scale. In Europe populations are increasingly digitally literate, which increases the feasibility of AF screening at scale. Before AF screening is implemented at scale, reproducible prospective evidence of benefit is required.

## Introduction

Atrial fibrillation (AF) is on the rise, and it is estimated that there will be 17.9 million cases across the European Union by 2060.^1^ Population-based systematic screening of atrial fibrillation for its early detection and treatment may reduce stroke and other adverse clinical events.^2^, ^3^, ^4^ The use of patient-centred non-invasive digital devices appears the most promising approach to make screening for AF feasible and acceptable to patients, as per evidence from European Society of Cardiology (ESC) AF guidelines.^3^, ^4^, ^5^ Yet, whilst it is understood that more AF is detected with this approach than with routine care,^6^, ^7^, ^8^ the target population and screening procedures to optimise recruitment and yield of incident AF, and whether this improves outcomes, is uncertain.

Previous systematic reviews of AF screening have provided an incomplete overview for systematic AF screening using non-invasive devices. Some have not provided a quantitative synthesis,^6^^,^^9^ others only explored yields of AF screening with a single time point strategy,^10^ one preceded the publication of several landmark randomised clinical trials (RCTs),^7^ and all precede studies exploring the use of risk prediction models for AF, including those developed with machine learning, to guide AF screening.^8^^,^^11^^,^^12^ Meta-analysis for outcomes of AF screening have included data from RCTs identifying AF through invasive long-term monitoring,^13^ which is not scalable or appropriate for the public and may detect a different AF phenotype.^14^ In addition, different approaches to invitation, consent and rhythm recording may impact on recruitment, use of rhythm monitoring devices, and yield but this has not been summarised across multiple studies.

To address this knowledge gap we performed a systematic review and meta-analysis to describe, amongst studies of systematic population-based AF screening with non-invasive devices, (i) the approaches to recruitment and consent, (ii) yields of AF, and (iii) outcomes.

## Methods

### Search strategy and selection criteria

We searched the Medline and Embase, through the Ovid platform in addition to Web of Science and Cochrane Library, from inception through 1st February 2025. We used a combination of keywords and subject headings related to AF and screening based on previous literature; the search was limited to the English language (Supplementary material).^7^^,^^8^^,^^10^ We completed forward and backward citation searching for included studies and previous systematic reviews. Duplicates were removed using Endnote’s duplicate identification strategy and then manually.

We included studies that prospectively investigated systematic non-invasive AF screening in an adult population, formalised using the PICO framework (Supplementary material).^15^ Systematic screening is when an entire population or stratum of a population is targeted screening.^16^ The inclusion criteria were as follows: (i) participants were adults (age ≥ 18 years) living in the community (patients with previous stroke or transient ischaemic attack were only considered if they constituted a proportion of the larger population), (ii) newly identified AF was differentiated from prevalent AF ], (iii) the diagnosis of AF needed to be confirmed using an electrocardiogram (ECG) (any number of leads) interpreted by an appropriately trained healthcare professional, and (iv) prospective systematic screening was employed, defined as screening carried out in all people over a certain age or in a particular sub-group, and no screening was defined as a passive approach towards the diagnosis of AF (that is, usual care). Studies were grouped into RCTs, where an intervention arm (systematic AF screening) and control arm (usual care) were reported, and prospective studies where an intervention arm (systematic AF screening) was reported alone without a comparator (termed ‘prospective cohort’ studies). Case series and review articles were excluded.

We uploaded records to a systematic review web application (Rayyan, Qatar Computing Research Institute).^17^ Four investigators (AW, MH, MF, KR) independently screened them for inclusion by title, abstract and full text and Supplementary materials. Disagreements were resolved by consultation with a fifth investigator (RN). This review was registered on PROSPERO (CRD42024572091) and informed by the PRISMA statement.^18^

Two investigators (AW and RN) independently extracted the data from the included studies, including pertaining to study and participant characteristics, and methods of recruitment, consent and rhythm recording. Meetings between team members were scheduled every two weeks over period of 3 months. Patients’ demographics such as age, gender profile, baseline co-morbidities, outcomes including hospitalisation, mortality was recorded for each study. Other data inputs included number of participants invited, consented, completed the study process and loss to follow up. Data on consenting methodology, inclusion, exclusion criteria, duration and modality of non-invasive screening for each study, was collated. Disagreements were discussed with HL and JW in monthly meetings. Three investigators (AW, KR, and RN) assessed risk of bias using the modified Cochrane collaboration’s risk of bias tool for RCTs,^19^ and the ROBINS-I tool for prospective cohort studies.^20^

### Data analysis

The co-primary outcome was the incidence of previously undiagnosed AF as the result of systematic screening (therefore the detection of incident AF), and outcomes reported after AF screening including all-cause death, cardiovascular hospitalisation, stroke, systemic embolism and bleeding. An intention-to-treat analysis was conducted, the denominator being all patients qualified to be screened who were eligible and consented. Prospective cohort studies with fewer than 100 patients in the intervention arm were not eligible for meta-analysis to improve precision. Incidence rates and relative risks were calculated and random effects meta-analysis performed to synthesise rates of AF in prospective studies and RCTs, respectively, and outcomes of AF in RCTs. Logit transformation was applied to random effects models of incidence rate as several observed proportions were close to 0. The prospective study subgroup included both single arm prospective cohorts and RCT populations where only the results of the intervention arm have been reported to date (thus precluding comparison to the control group). Statistical heterogeneity was evaluated with Cochran’s Q-statistic and quantified with the I^2^ statistic for synthesised estimates. The results of meta-analysis are presented in forest plots.

Subgroup analyses related to AF yield were carried out by: (i) method of participant selection (age, age + stroke risk factor, age + biomarker, AF risk stratification algorithm), (ii) modality of monitoring (intermittent ECG, continuous ECG, intermittent photoplethysmography (PPG), continuous PPG, or single time point ECG; definitions in Supplementary material). In addition, potential sources of heterogeneity including mean age, sex, modality of monitoring, and selection method were tested in univariate meta-regression models of prospective studies and RCTs where the number of studies were 10 or more. For categorical covariates, only levels with 4 or more studies were included in the meta-regression to ensure sufficient statistical power.

Sensitivity analyses were conducted to exclude studies at high risk of bias and to explore effect of publication year (excluding studies published before 2010). Funnel plots and Egger’s test for asymmetry in distribution of standard errors were examined to assess possible publication bias and outliers among RCTs or prospective studies. We considered p < 0.05 to be statistically significant where the total number of studies in the sample were 10 or more. All analyses were conducted using R software (version 4.2.1).^21^

### Role of funding source

The funder had no role in the design, data collection, data analysis, data interpretation or writing of the report.

## Results

**Fig. 1:**
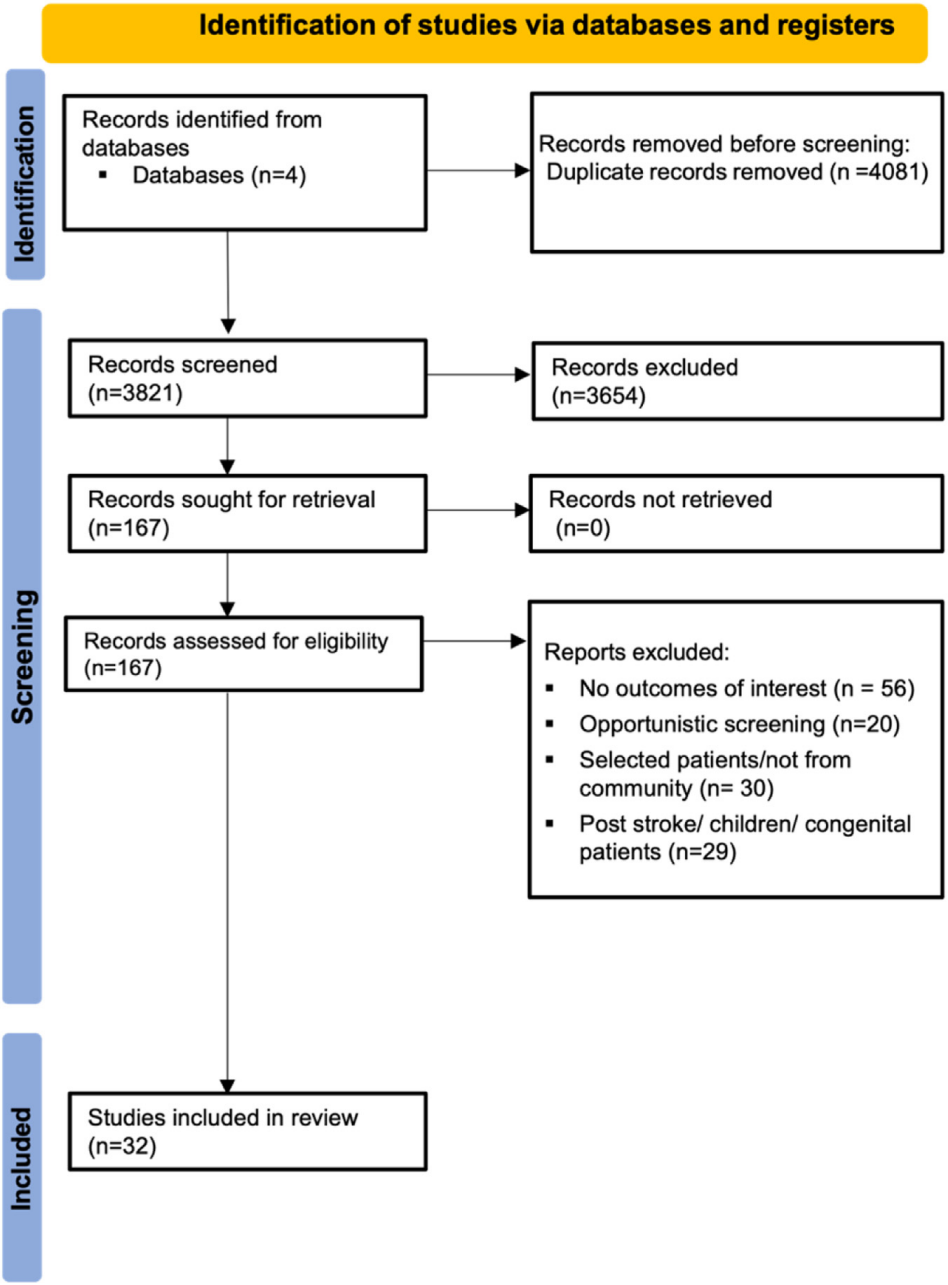
The PRISMA flowchart of studies included for systematic review and meta-analysis.

We identified 3821 unique records, reviewed 167 full-text reports and included 32 studies (Fig. 1). Excluded studies that met a number of inclusion criteria are reported in the Supplementary material. Included studies reported 8 RCTs^11^^,^^22^, ^23^, ^24^, ^25^, ^26^, ^27^, ^28^ and 24 prospective cohort studies^12^^,^^29^, ^30^, ^31^, ^32^, ^33^, ^34^, ^35^, ^36^, ^37^, ^38^, ^39^, ^40^, ^41^, ^42^, ^43^, ^44^, ^45^, ^46^, ^47^, ^48^, ^49^, ^50^ or reports of only the intervention arm of a RCT, encompassing 11 countries (Supplementary Tables S1 and S2). Studies included a total of 735,542 patients with a mean age of 70.4 (standard deviation [SD] 11.6) years, 36% women, and mean CHA_2_DS_2-_VASc 2.9 (SD 0.64).

Twenty one studies reported the number of invitations sent and the number who consented to participate in AF screening (Supplementary Tables S3 and S4). Twenty one used written consent procedures, nine used digital consent procedures, and two consented eligible participants in-person. Participants in studies that implemented digital consent procedures, compared to written consent procedures, were younger (mean age 58.9 v 74.9, p = 0.03). Consent rates were higher overall in studies employing digital procedures (69.8%, 711,135/1,017,779) compared with written procedures (37.1%, 46,227/124,481).

Across the studies, 22% (7/32) conducted screening through a consumer-owned device, 9% (3/32) provided the rhythm recording device in person, and 69% (22/32) sent the device to the patient for rhythm recording. The proportion of participants who used the device of those invited was 89%, 75%, and 97% for consumer-owned devices, in-person receipt, and remote receipt, respectively. Five studies used a two-step process of AF screening with an initial period of PPG followed by confirmation with an ECG recorder for those with an irregular notification. In these studies, of participants who received an irregular pulse notification from PPG, 4.1% attended follow on ECG monitoring or clinic review.

**Fig. 2:**
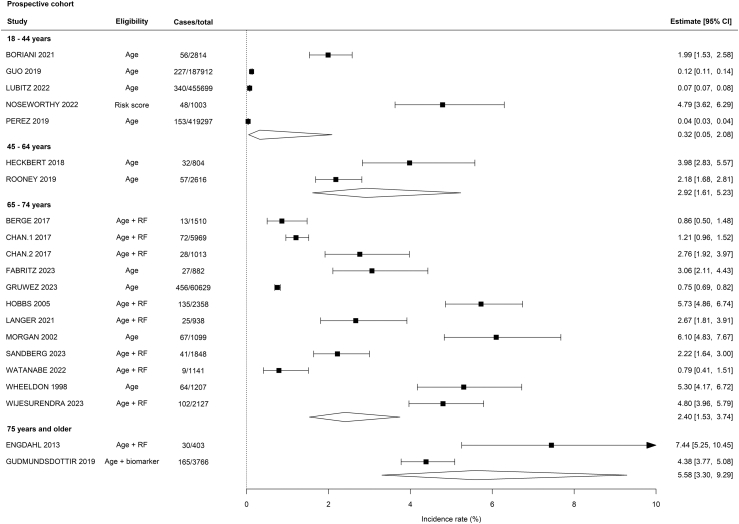
Forest plot of incidence rate of new atrial fibrillation diagnosis in prospective cohort studies or reports of the intervention arm alone from a randomised clinical trial of AF, stratified by median age of participants.

**Fig. 3:**
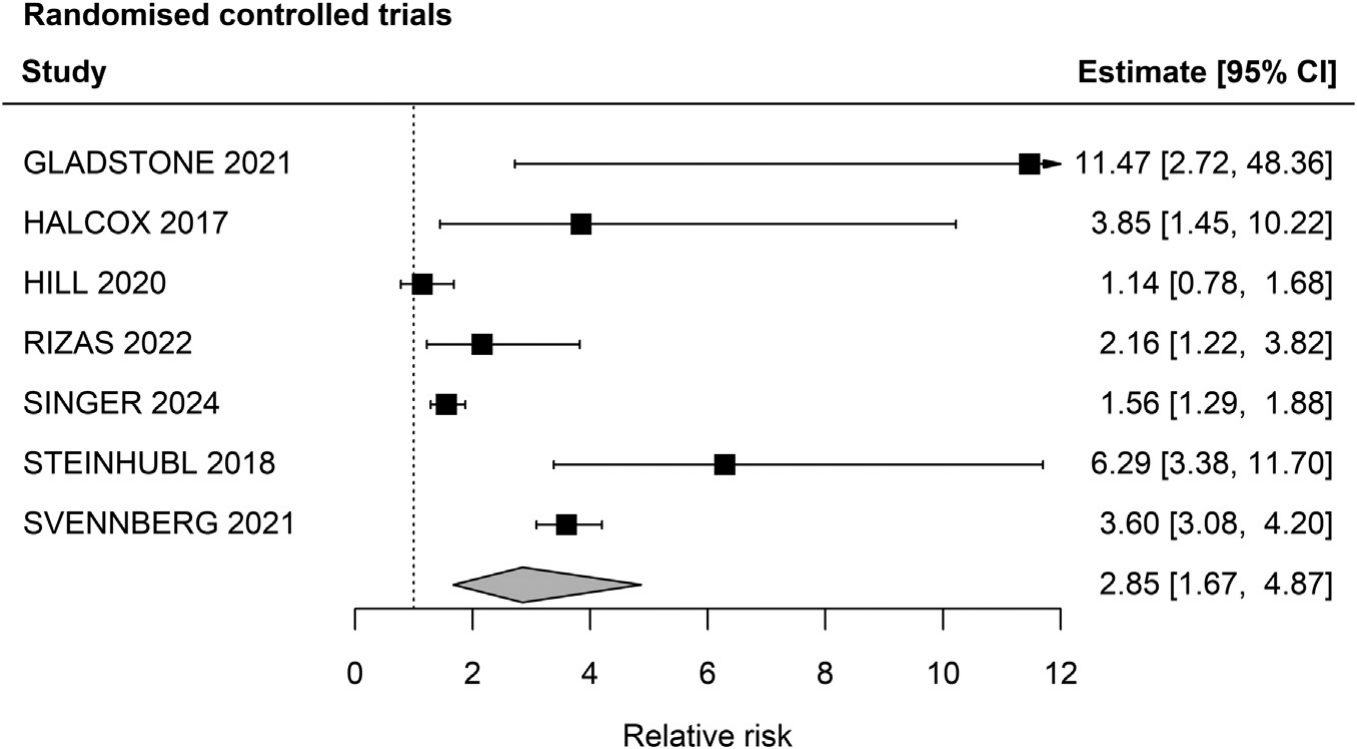
Forest plot of the relative risk for new atrial fibrillation diagnoses in randomised clinical trials of AF screening comparing the intervention and control arm.

Overall, the incidence rate for new AF in prospective cohorts was 1.53% (95% CI 0.81–2.89) (Supplementary Fig. S1) with an increasing gradient of AF detection with increasing median age of the participants (Fig. 2). In RCTs the pooled relative risk between intervention arms and routine care was 2.22 (95% CI 1.41–3.50) (Fig. 3).

**Fig. 4:**
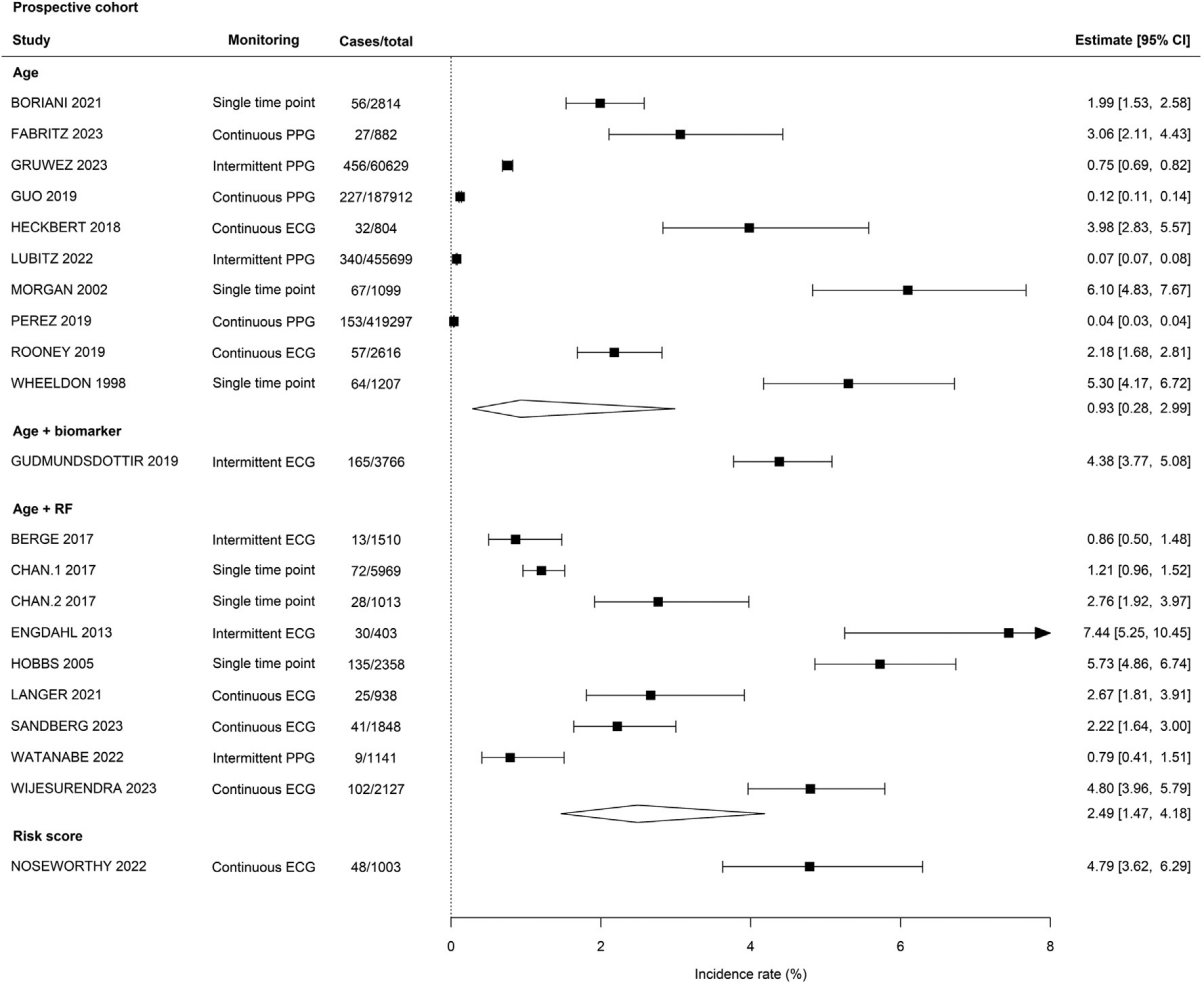
Forest plot of incidence rate of new atrial fibrillation diagnosis in prospective cohort studies or reports of the intervention arm alone from a randomised clinical trial of AF screening stratified by methods of selection of participants.

Fourteen studies used age alone to identify the target population for screening, three used stroke risk, 12 selected participants based on age plus at least one stroke risk factor, and three were guided by predicted AF risk. Meta-regression analysis in prospective cohorts demonstrated for every year increase in mean age of the population the diagnosis rate for incident AF increased (IRR 1.11, 95% CI 1.08–1.14) (Table 1), and the rate of AF detection was seven-fold higher in studies with a minimum eligibility age of 65 years compared to those inclusive of younger participants (IRR 7.66, 95% CI 1.93–30.35). Enriching the target population based on NT-proBNP level (IR 4.38%, 95% CI 3.77–5.08) or a risk score classification (4.79%, 95% CI 3.62–6.29) was associated with a higher yield than using age alone (0.93%, 95% CI 0.28–2.99) (Fig. 4). However, on meta-regression selecting the population based on age and stroke risk factors did not lead to a statistically higher yield of newly diagnosed AF compared with age alone (IRR 2.68, 95% CI 0.68–10.52) (Table 1).

**Table 1:** Meta-regression analysis of prospective cohorts of systematic screening for AF with non-invasive devices.

	Studies, N	Incidence rate (95% CI)	p value
Mean age, years	20	1.11 (1.08–1.14)	<0.001
Males, %	19	0.94 (0.89–0.99)	<0.05
CHAD2VASC score	16	2.73 (1.53–4.86)	<0.001
**Type of monitoring**			
Continuous ECG	6	Ref	
Single time point	6	1.01 (0.53–1.92)	0.970
**Minimum age criterion**			
18–44 years	5	Ref	
65–74 years	12	7.66 (1.93–30.35)	<0.01
**Eligibility**			
Age	10	Ref	
Age + RF	9	2.68 (0.68–10.52)	0.157

Of included studies, seven employed intermittent monitoring for AF detection, 10 used continuous monitoring, seven used single time point ECG assessment, three used intermittent PPG, and five used continuous PPG for diagnosis of AF. The use of intermittent PPG monitoring was associated with lower yields of diagnosis of incident AF in prospective cohorts (IR 0.35%, 95% CI 0.07–1.61) than continuous ECG (3.28%, 95% CI 2.41–4.44; Supplementary Fig. S2). Whilst screening with a single time point methodology was not associated with lower yields of detection of incident AF compared to more prolonged ECG monitoring when all prospective studies were included (3.30%, 95% CI 1.93–5.60) (Supplementary Fig. S2), sensitivity analysis where studies before 2010 were excluded demonstrated that this was skewed by older studies (1.85%, 95% CI 1.15–2.96) (Supplementary Fig. S3). Funnel plots did not demonstrate small studies effect for prospective cohort studies (Egger’s p-value 0.245) (Supplementary Fig. S4).

**Fig. 5:**
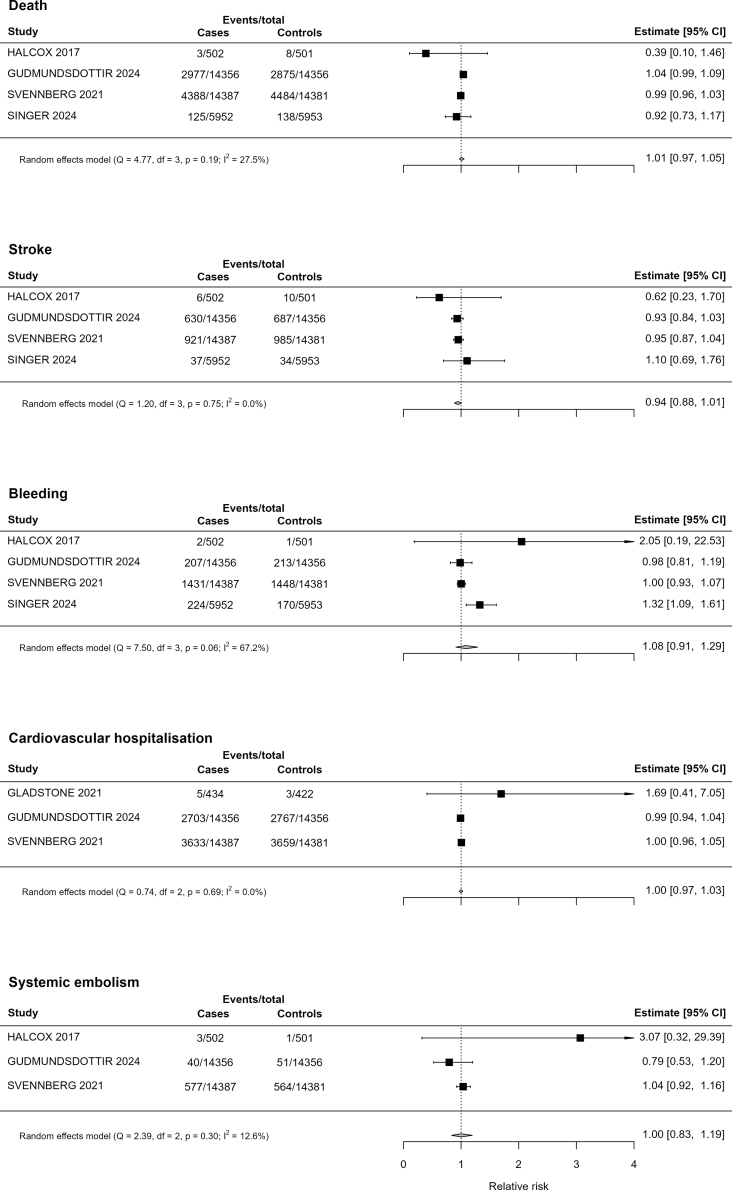
Forest plot of outcomes of systematic screening for atrial fibrillation using non-invasive devices.

Systematic screening for AF with non-invasive devices was not found to have an effect on outcomes including all-cause death (RR 1.01, 95% CI 0.97–1.05), cardiovascular hospitalisation (1.00, 95% CI 0.97–1.03), stroke (0.95, 95% CI 0.87–1.04), systemic embolism (1.00, 95% CI 0.83–1.19), or bleeding (1.08, 95% CI 0.91–1.29) (Fig. 5). Funnel plots demonstrated no evidence of small studies effect (Supplementary Fig. S5).

Overall, the risk of bias was moderate for both prospective studies (28% low, 64% moderate, 8% high) and RCTs (75% low and 25% moderate) (Supplementary Figs. S6 and S7). Removing studies at high risk of bias made little difference to the overall incidence rate and pattern observed in prospective studies (Supplementary Fig. S8).

## Discussion

This systematic review and meta-analysis quantitatively synthesises summary data from over 700,000 individuals who participated in AF screening studies. Whilst older age was associated with an increased yield of diagnosis of incident AF during screening, the diagnostic yield may be further enhanced through the use of an AF risk score or NT-proBNP. Digital procedures for recruitment and consent of participants are feasible but hitherto most often used in younger, less comorbid populations. The remote delivery of ECG monitoring devices is associated with high progression to participation in screening, but incorporating multiple steps to achieve an AF diagnosis (e.g., PPG followed by ECG) leads to an attrition in participation. The present evidence base does not demonstrate an effect on rate of stroke, death, or cardiovascular hospitalisation from systematic screening for AF using non-invasive devices.

Previous systematic reviews have demonstrated that systematic is more effective at detecting new AF than opportunistic screening and routine care,^7^^,^^51^ and that increasing participant age is associated with an higher yield of AF detection during screening.^10^^,^^52^ Systematic screening approach is undergoing validation through current recruitment in two large prospective studies, FIND-AF^53^ and CONSIDERING-AF.^54^ Nonetheless, previous reviews have not considered more sophisticated methods to target AF screening beyond age alone. Our data, incorporating contemporary studies, suggests that further refining the ‘first filter’ for AF screening beyond age alone can increase yield of incident AF detection, though our certainty is limited by the paucity of completed studies investigating this.^55^

Furthermore, previous reviews have summarised AF detection yield though not considered the logistical steps involved in the clinical screening pathway. We have found that digital and remote recruitment and rhythm monitoring procedures are feasible and lead to high numbers of participants across multiple studies, reinforcing the validity and potential of this approach. Many decentralised studies of digital recruitment through consumer-owned devices led to recruitment of younger and less comorbid patients^34^^,^^35^^,^^37^ who, as expected, had lower rates of undiagnosed AF. Notably, these trials had significant loss between the initial PPG alert step and the confirmatory diagnostic step. The recruited younger less comorbid participants may have been less naturally medicalised, may demonstrate an imbalance between traditional and digital engagement in research studies and clinical care, and may place a lower importance on receiving a formal diagnosis if they are self-aware that they are at lower stroke risk. However, recent studies have also shown that fully digital self-screening studies are feasible in older patients in European countries with stroke risk factors through social media and smartphones.^22^^,^^50^

Notably, machine learning-derived risk scores for incident AF have been developed within routine health care (electronic health record or insurance) datasets,^56^, ^57^, ^58^ and thus are amenable to be implemented at scale within existing health structures. Whilst the use of biomarkers may also identify individuals more likely to have AF diagnosed during screening,^59^^,^^60^ the requirement for an additional appointment and investigation as part of risk stratification would place extra burden on both healthcare professionals, patients, and incur healthcare expenditure. By contrast, the use of algorithms within routinely collected data could act as a less costly ‘data’ biomarker for AF risk, and two large prospective studies are investigating this approach to guide AF screening.^53^^,^^54^ These studies utilise digital consent procedures and remote delivery and conduct of ECG monitoring that our study suggests may be the optimum an AF screening pathway.

Improving yield of new AF diagnosed through screening has hitherto been seen as an important component of making AF screening effective at improving health outcomes and being cost-effective. However, ongoing research is required to address the issues of the effectiveness and safety of treatment of screen-detected AF, and the costs of widespread use of ECG monitoring and prescription of oral anticoagulation. Our results concord with a previous meta-analysis that there is insufficient evidence as yet that screening for AF improves clinical outcomes,^14^ in spite of two further RCTs being published this year.^27^^,^^61^ There is mounting evidence that screen-detected AF may not bear a similar stroke and mortality risk profile to clinically detected AF, particularly when AF screening is done at a higher intensity than single-timepoint.^62^ Furthermore in the population the incidence of stroke is falling, meaning that declines in stroke risk from AF screening and treatment are lower than expected.^63^ A trial sequential analysis has suggested that an optimal sample size of a total of 103,454 participants randomized is required to demonstrate a benefit of stroke.^14^ The number of patients randomised included in this meta-analysis for the stroke outcome is only 70,388. Thus it may be that trials that are ongoing will help definitively answer whether AF screening is beneficial to clinical outcomes, but the effect may be smaller than expected.

Thus for screening for AF to be effective, the intervention and outcome may need to extend beyond stroke. Individuals identified at risk of AF have also been demonstrated to be at risk of other complications of AF such as heart failure.^64^ Thus, the utility of risk-guided AF screening may extend to risk factor modification in those with ‘pre-AF’,^65^ those who show signs of deterioration in left ventricular systolic function, as well as the identification of those who may benefit from longitudinal screening.^66^

There are limitations of this meta-analysis and its findings. First, we utilised study-level rather than individual patient data. Second, it was not feasible or statistically prudent to synthesise prospective cohort studies and RCTs together and so the results are reported separately.^67^ Third, heterogeneity of effect across studies was expected, incorporated for by use of random effects meta-analysis, and explored with sensitivity analyses, tests of publication bias and meta-regression but was still substantial (Supplementary Table S6). This is likely due to differences in study participants, interventions, outcomes, and usual care (clinical heterogeneity) as well as variation in study designs and risks of bias (methodological heterogeneity) (Supplementary Table S5). Fourth, the smaller number of RCTs precluded robust assessment of different approaches to identify the target population on AF yield in this study design. Fifth the large number of participants in decentralised studies that employed an initial PPG step but had low rates of diagnoses and follow up amongst younger less comorbid patients may have influenced the results for AF yield.

This meta-analysis of over 700,000 individuals who participated in systematic AF screening studies with a non-invasive device demonstrates that the yield for the diagnosis of incident AF can be enhanced through the selection of a higher risk population, and that patients may be recruited and screened remotely and digitally. The pooled estimates fail to demonstrate that screening for AF with a non-invasive device reduces death, cardiovascular hospitalisation, or stroke. Screening studies of AF employing alternative risk stratifications and outcome measures are required.

## Contributors

AW identified potential records through database searches, extracted data from included studies, generated Supplementary material, performed Cochrane and ROBINS risk of bias for RCT’s and prospective studies respectively and contributed towards generation of first draft.

RN conceived the idea, extracted data from included studies, generated the first and second draft, reviewed Supplementary material and assessed risk of bias.

MH, MF, KR and UN contributed towards risk of bias, review and editing of Supplementary material. Statistical analysis and generation of Forrest plots was undertaken by HL and JW.

Remaining authors (TJ, AB, CW, KK, RV, AJC, EV, GYL, BF) contributed towards reviewing and editing the manuscript. CPG reviewed and edited all the versions of manuscripts and overall provided guidance and direction. All authors were responsible for the decision to submit the manuscript.

## Data sharing statement

Data are available on reasonable request. Technical appendix, statistical code and dataset are available from the corresponding author at r.nadarajah@leeds.ac.uk.

## Declaration of interests

All authors have completed the ICMJE uniform disclosure form at www.icjme.org/col_disclosure.pdf and declare: HL is currently employed by NovoNordisk. TJ has received authors payment for Medicine Journal; honoraria from speaking at meetings for Janssen. CW has received research grants from British Heart Foundation. KK has received research grants from AstraZeneca, Boehringer, Ingelheim, Lilly, MSD, NovoNordisk, Sanofi, Servier, Oramed Pharmaceuticals, Roche, Daiichi-Sankyo and Applied Therapeutic; consulting fees from Amgen, AstraZeneca, Bristol Myers Squibb, Boehringer Ingelheim, Lilly, NovoNordisk, Sanofi, Servier, Pfizer, Roche, Daiichi-Sankyo, Embecta and Nestle Health Science; honoraria from speaking at meetings and educational events at Amgen, AstraZeneca, Bristol Myers Squibb, Boehringer, Ingelheim, Lilly, NovoNordisk, Sanofi, Servier, Pfizer, Roche, Daiichi-Sankyo, Embecta and Nestle Health Science. AJC receives consultations fees from Bayer, Pfizer, Daiichi Sankyo, Acesion, InCarda, Abbott, Boston Scientific, Medtronic, Huya Biosceince and Biosense Webster; honoraria from speaking at meetings and educational events at Sanofi, Menarini and Boston Scientific. ES has received grants for research from Stockholm County Council, Ake Wiberg Foundation, Swedish Heart foundation and CIMED Heart and Lung foundation; consulting fees from Bayer, Bristol-Myers and Squibb-Pfizer, Boehringer- Ingelheim, Johnson & Johnson, Merck Sharp & Dohme and Abbott; honoraria from meetings and educational events held at Bayer, Bristol- Myers Squibb-Pfizer, Boehringer-Ingelheim, Johnson &Johnson, Merck Shark & Dohnme and Abbott. BF has received speaker fees and consultancy fees from BMS/Pfizer Alliance, speaker fees from Omron; Investigator initiated study grant to BMS/Pfizer Alliance. CPG has received grants for research from Alan Turing Institute, British Heart Foundation, National Institute for Health Research, Horizon 2020, Abbott Diabetes, Bristol Myers Squibb and European Society of Cardiology; consulting fees from AI Nexus, Astrazeneca, Bayer, Amgen, Bristol Myers Squibb, Boehringer-Ingelheim, CardioMatics, Chiesi, Daiichi Sankyo General Practitioner Research Institute (GPRI), Menarini, Novartis, iRhythm, Organon and The Phoenix Group; honoraria for speaking at meetings and educational events from Astrazeneca, Boston Scientific, Menarini, Novartis, Raisio Group Wondr Medical and Zydus; support for attending meetings from Astrazeneca; participated on advisory board for DANBLCOK and TARGET CTCA trials; chaired role of deputy editor of European Heart Journal (EHJ) Quality of Care and Clinical Outcomes journal and committee member for NICE Indicator Advisory Committee and ESC Quality Indicator Committee; has stock option in CardioMatics; in receipt of KOSMOS device. All other authors declare no competing interests, or activities that could appear to have influenced the submitted work.
