# Supplementary material for: Systematic screening for atrial fibrillation with non-invasive devices: a systematic review and meta-analysis
Source: Lancet Reg Health Eur. 2025 Apr 11;53:101298. doi: 10.1016/j.lanepe.2025.101298 (PMC12018576; doi:10.1016/j.lanepe.2025.101298)
Supplement: Supplementary Figs. S1–S8 and Tables S1–S6 [file mmc1.docx]

**Supplementary appendix**

Systematic screening for atrial fibrillation with non-invasive devices: a systematic review and meta-analysis

Ali Wahab*, Ramesh Nadarajah*, Harriet Larvin, Maryum Farooq, Keerthenan Raveendra, Mohammad Haris, Umbreen Nadeem, Tobin Joseph, Asad Bhatty, Chris Wilkinson, Kamlesh Khunti, Rajesh Vedanathan, A John Camm, Emma Svennberg, Gregory YH Lip, Ben Freedman, Jianhua Wu, Chris P Gale

Contents

[**Section 1**- OVID EMBASE Search Strategy 2](#_Toc190828683)

[**Section 2**- Medline Search Strategy 5](#_Toc190828684)

[**Section 3**: Cochrane Library Search Strategy 8](#_Toc190828685)

[**Section 4** – Web of Science Search Strategy 10](#_Toc190828686)

[**Section 5:** Definitions 13](#_Toc190828687)

[**Figure 1:** Forest plot of incidence rate of new atrial fibrillation diagnosis in prospective cohort studies or reports of the intervention arm alone from a randomised clinical trial of AF. 15](#_Toc190828688)

[**Figure 2:** Forest plot of incidence rate of new atrial fibrillation diagnosis in prospective cohort studies or reports of the intervention arm alone from a randomised clinical trial of AF, stratified by monitoring type. 16](#_Toc190828689)

[**Figure 3**: Forest plot of incidence rate of new atrial fibrillation diagnosis in prospective cohort studies or reports of the intervention arm alone from a randomised clinical trial of AF, stratified by monitoring type limited to studies published after 2010. 17](#_Toc190828690)

[**Figure 4:** Funnel plot for results of yield from prospective cohort studies 18](#_Toc190828691)

[**Figure 5**- Funnel plot of for results of clinical outcomes from randomised clinical trials 19](#_Toc190828692)

[**Figure 6**- Risk of Bias for prospective cohort studies. 20](#_Toc190828693)

[**Figure 7**- Risk of Bias for RCT’s 21](#_Toc190828694)

[**Figure 8:** Forrest plot after exclusion of high ROB prospective studies 22](#_Toc190828695)

[**Table 1**- Baseline characteristics of RCT 23](#_Toc190828696)

[**Table 2**- Baseline characteristics of prospective cohort studies. 24](#_Toc190828697)

[**Table 3**: Characteristics of recruitment and protocol adherence (RCT’s) 27](#_Toc190828698)

[**Table 4:** Characteristics of recruitment and protocol adherence (observational studies) 28](#_Toc190828699)

[**Table 5:** Baseline characteristics of all studies 30](#_Toc190828700)

[**Table 6**- Cochran's Q, degrees of freedom and I^2 measures of statistical heterogeneity within subgroup analyses 32](#_Toc190828701)

# Section 1- OVID EMBASE Search Strategy

| **#** | **Searches** | **Results** |
| --- | --- | --- |
| 1 | exp atrial fibrillation/ | 143475 |
| 2 | ((atrial or atria or atrium or auricular) adj3 fibrillat*).ti,ab. | 179794 |
| 3 | AF.ti,ab. | 108556 |
| 4 | or/1-3 [atrial fibrillation] | 253401 |
| 5 | (diagnos* adj3 (performance* or accurac* or utilit* or value* or efficien* or effectiveness)).ti,ab. | 274888 |
| 6 | Mass screening/ | 69216 |
| 7 | ((targeted or opportunistic) adj2 (detect* or screen*)).ti,ab. | 11528 |
| 8 | point of care.ti,ab. | 47683 |
| 9 | electronic health record/ | 48166 |
| 10 | electronic medical record system/ | 3010 |
| 11 | ("electronic patient record*" or "electronic medical record*" or "electronic health record*" or "computeri?ed patient record*" or "computeri#ed medical record*" or "computeried health record*" or EHR or EPR).ti,ab. | 150768 |
| 12 | ((portable or ambulatory or monitor* or lead* or handheld or hand held or daily or longterm or short-term or strap* or device*) adj3 (ECG* or EKG* or electrocardio*)).ti,ab. | 46623 |
| 13 | ((ECG* or EKG* or electrocardio*) adj2 (assess* or check* or monitor* or detect* or screen* or diagnos* or measure*)).ti,ab. | 31033 |
| 14 | (iECG* or Holter*).ti,ab. | 22593 |
| 15 | ((ambulatory or event) adj monitor*).ti,ab. | 5982 |
| 16 | *electrocardiography/ or ambulatory electrocardiography/ | 63751 |
| 17 | (ILR* or loop record*).ti,ab. | 3974 |
| 18 | ((heart or cardiac) adj monitor*).ti,ab. | 5727 |
| 19 | (pulse adj2 (assess* or check* or monitor* or detect* or screen* or diagnos* or measure* or palpation*)).ti,ab. | 11856 |
| 20 | (pulse oximetr* adj device*).ti,ab. | 59 |
| 21 | Pulse rate/ | 62560 |
| 22 | ((blood pressure or BP) adj2 (assess* or check* or monitor* or detect* or screen* or diagnos* or measure*)).ti,ab. | 78454 |
| 23 | Blood Pressure Monitor/ or Blood Pressure Monitoring/ | 67407 |
| 24 | (AliveCor or MyDiagnostic*).ti,ab. | 335 |
| 25 | (Microlife or WatchBP or "watch BP").ti,ab. | 349 |
| 26 | (Heartscan or Zenicor or AliveECG or Kardia*).ti,ab. | 399 |
| 27 | (photoplethysmograph* or PPG).ti,ab. | 10867 |
| 28 | (wearable adj2 (technology or device* or sensor* or ECG or EKG or electrocardio*)).ti,ab. | 17365 |
| 29 | (smartwatch* or smart watch* or Applewatch* or Apple watch* or wrist watch* or wristwatch* or fitness band* or fitness tracker* or smartphone* or smart phone* or mobile phone*).ti,ab. | 51311 |
| 30 | (wearable adj2 (technology or device* or sensor* or ECG or EKG or electrocardio*)).ti,ab. | 17365 |
| 31 | or/6-30 [ point of care tests used to detect AF or screening or diagnosis effectiveness terms] | 674245 |
| 32 | randomized controlled trial/ | 837901 |
| 33 | random*.ti,ab. | 2113492 |
| 34 | factorial*.ti,ab. | 50583 |
| 35 | (crossover* or cross over*).ti,ab. | 133109 |
| 36 | ((doubl* or singl*) adj blind*).ti,ab. | 291615 |
| 37 | (assign* or allocat* or volunteer* or placebo*).ti,ab. | 1346023 |
| 38 | crossover procedure/ | 79391 |
| 39 | single blind procedure/ | 55767 |
| 40 | double blind procedure/ | 224517 |
| 41 | or/32-40 [RCTs] | 3123585 |
| 42 | Prospective studies/ | 828016 |
| 43 | (prospective and (study or studies or review or analys* or cohort* or data)).ti,ab. | 1122625 |
| 44 | or/42-43 [prospective studies] | 1387690 |
| 45 | 41 or 44 [RCTS or prospective studies] | 4208177 |
| 46 | 4 and 31 and 45 | 5934 |
| 47 | letter/ or letter.pt. | 1342536 |
| 48 | note.pt. | 996750 |
| 49 | editorial.pt. | 816503 |
| 50 | case study/ or case report/ | 3216548 |
| 51 | (letter or comment*).ti. | 257479 |
| 52 | or/47-51 [excluded publication types] | 6106486 |
| 53 | randomized controlled trial/ or random*.ti,ab. | 2235916 |
| 54 | 52 not 53 | 6043916 |
| 55 | animals/ not humans/ | 1522658 |
| 56 | nonhuman/ | 7813476 |
| 57 | exp Animal Experiment/ | 3229408 |
| 58 | exp Experimental Animal/ | 896635 |
| 59 | animal model/ | 1819455 |
| 60 | exp Rodent/ | 4637763 |
| 61 | (rat or rats or mouse or mice).ti. | 1856463 |
| 62 | or/55-61 [animal studies] | 10670021 |
| 63 | 46 not 52 | 5800 |
| 64 | 63 not 62 [final results excluding animals or certain excluded publication types] | 5630 |
| 65 | limit 64 to dc=19900101-20250201 | 6130 |

# Section 2- Medline Search Strategy

| **#** | **Searches** | **Results** |
| --- | --- | --- |
| 1 | exp atrial fibrillation/ | 75324 |
| 2 | ((atrial or atria or atrium or auricular) adj3 fibrillat*).ti,ab. | 97808 |
| 3 | AF.ti,ab. | 56915 |
| 4 | or/1-3 [atrial fibrillation] | 129523 |
| 5 | (diagnos* adj3 (performance* or accurac* or utilit* or value* or efficien* or effectiveness)).ti,ab. | 187741 |
| 6 | Mass screening/ | 118553 |
| 7 | ((targeted or opportunistic) adj2 (detect* or screen*)).ti,ab. | 7881 |
| 8 | point of care.ti,ab. | 35535 |
| 9 | electronic health record/ | 28942 |
| 10 | Medical Records Systems, Computerized/ | 19168 |
| 11 | ("electronic patient record*" or "electronic medical record*" or "electronic health record*" or "computeri?ed patient record*" or "computeri#ed medical record*" or "computeried health record*" or EHR or EPR).ti,ab. | 91961 |
| 12 | ((portable or ambulatory or monitor* or lead* or handheld or hand held or daily or longterm or short-term or strap* or device*) adj3 (ECG* or EKG* or electrocardio*)).ti,ab. | 27302 |
| 13 | ((ECG* or EKG* or electrocardio*) adj2 (assess* or check* or monitor* or detect* or screen* or diagnos* or measure*)).ti,ab. | 18665 |
| 14 | (iECG* or Holter*).ti,ab. | 12233 |
| 15 | ((ambulatory or event) adj monitor*).ti,ab. | 3946 |
| 16 | *electrocardiography/ or electrocardiography, ambulatory/ | 81059 |
| 17 | (ILR* or loop record*).ti,ab. | 1894 |
| 18 | ((heart or cardiac) adj monitor*).ti,ab. | 3062 |
| 19 | (pulse adj2 (assess* or check* or monitor* or detect* or screen* or diagnos* or measure* or palpation*)).ti,ab. | 8479 |
| 20 | (pulse oximetr* adj device*).ti,ab. | 43 |
| 21 | Pulse/ | 17106 |
| 22 | ((blood pressure or BP) adj2 (assess* or check* or monitor* or detect* or screen* or diagnos* or measure*)).ti,ab. | 48360 |
| 23 | Blood Pressure Monitors/ or Blood Pressure Monitoring, Ambulatory/ | 14278 |
| 24 | (AliveCor or MyDiagnostic*).ti,ab. | 144 |
| 25 | (Microlife or WatchBP or "watch BP").ti,ab. | 144 |
| 26 | (Heartscan or Zenicor or AliveECG or Kardia*).ti,ab. | 584 |
| 27 | (photoplethysmograph* or PPG).ti,ab. | 7611 |
| 28 | (wearable adj2 (technology or device* or sensor* or ECG or EKG or electrocardio*)).ti,ab. | 15817 |
| 29 | (smartwatch* or smart watch* or Applewatch* or Apple watch* or wrist watch* or wristwatch* or fitness band* or fitness tracker* or smartphone* or smart phone* or mobile phone*).ti,ab. | 40493 |
| 30 | (wearable adj2 (technology or device* or sensor* or ECG or EKG or electrocardio*)).ti,ab. | 15817 |
| 31 | or/6-30 [ point of care tests used to detect AF or screening or diagnosis effectiveness terms] | 514373 |
| 32 | randomized controlled trial.pt. | 618008 |
| 33 | controlled clinical trial.pt. | 95579 |
| 34 | randomi#ed.ab. | 779935 |
| 35 | placebo.ab. | 250234 |
| 36 | randomly.ab. | 438150 |
| 37 | clinical trials as topic.sh. | 202832 |
| 38 | trial.ti. | 314193 |
| 39 | or/32-38 [RCTs] | 1663529 |
| 40 | Prospective studies/ | 692996 |
| 41 | (prospective and (study or studies or review or analys* or cohort* or data)).ti,ab. | 721716 |
| 42 | or/40-41 [prospective studies] | 989150 |
| 43 | 39 or 42 [RCTS or prospective studies] | 2446705 |
| 44 | 4 and 31 and 43 | 2752 |
| 45 | letter/ | 1263286 |
| 46 | editorial/ | 699240 |
| 47 | news/ | 225965 |
| 48 | exp historical article/ | 412158 |
| 49 | Anecdotes as Topic/ | 4747 |
| 50 | comment/ | 1038684 |
| 51 | case reports/ | 2418771 |
| 52 | (letter or comment*).ti. | 201988 |
| 53 | or/45-52 | 5117405 |
| 54 | randomized controlled trial/ or random*.ti,ab. | 1665931 |
| 55 | 53 not 54 | 5083060 |
| 56 | animals/ not humans/ | 5206979 |
| 57 | exp Animals, Laboratory/ | 966821 |
| 58 | exp Animal Experimentation/ | 10524 |
| 59 | exp Models, Animal/ | 656115 |
| 60 | exp Rodentia/ | 3626353 |
| 61 | (rat or rats or mouse or mice).ti. | 1473909 |
| 62 | or/55-61 | 11231350 |
| 63 | 44 not 62 [search results without animal studies or letters or commentaries etc.] | 2715 |
| 64 | limit 63 to dt=19900101-20250201 | 2759 |

# Section 3: Cochrane Library Search Strategy

| **#** | **Searches** | **Results** |
| --- | --- | --- |
| 1 | MeSH descriptor: [Atrial Fibrillation] explode all trees and with qualifier(s): [diagnosis - DI] | 7381 |
| 2 | (atria* or atrium or auricular) NEAR/3 (performance or accurac* or utilit* or value* or efficien* or effectiveness):ti,ab | 250 |
| 3 | AF:ti,ab | 9787 |
| 4 | MeSH descriptor: [Mass Screening] explode all trees | 6006 |
| 5 | (diagnos*) NEXT (performance* or accurac* or utilit* or value* or efficiency* or effectiveness*):ti,ab | 6094 |
| 6 | (targeted or opportunistic) NEAR/3 (detect* or screen*):ti,ab | 280 |
| 7 | point-of-care:ti,ab | 2837 |
| 8 | MeSH descriptor: [Electronic Health Records] explode all trees | 900 |
| 9 | MeSH descriptor: [Medical Records Systems, Computerized] explode all trees | 1215 |
| 10 | electronic patient record or electronic medical record or electronic health record or computeri*ed patient record* or computeri*ed health record* or EHR or EPR):ab,ti | 6601 |
| 11 | (portable or ambulatory or monitor* or lead* or handheld* or "hand held" or daily or "long term" or "short term" or strap or device) NEAR (ECG* or EKG or electrocardio*):ti,ab | 9678 |
| 12 | (ECG* or EKG or electrocardio*) NEAR (assess* or check or monitor* or detect* or screen* or diagnos* or measure*):ti,ab | 10600 |
| 13 | iECG or holter:ti,ab | 3187 |
| 14 | (ambulatory or event) NEXT (monitor*):ti,ab | 1227 |
| 15 | MeSH descriptor: [Electrocardiography] explode all trees | 10602 |
| 16 | MeSH descriptor: [Electrocardiography, Ambulatory] explode all trees | 1395 |
| 17 | ILR*:ti,ab | 442 |
| 18 | "loop record":ti,ab | 0 |
| 19 | (heart or cardiac) NEXT (monitor*):ti,ab | 908 |
| 20 | (pulse) NEXT (assess* or check or monitor or detect* or screen* or diagnos* or measure* or palpation*):ti,ab | 251 |
| 21 | (pulse oximetr* NEXT device):ti,ab | 69 |
| 22 | MeSH descriptor: [Pulse] explode all trees | 1576 |
| 23 | MeSH descriptor: [Blood Pressure Monitors] explode all trees | 185 |
| 24 | MeSH descriptor: [Blood Pressure Monitoring, Ambulatory] explode all trees | 1974 |
| 25 | ("blood pressure") NEXT (assess* or check or monitor or detect* or screen* or diagnos* or measure*):ti,ab | 5134 |
| 26 | aliveCOR:ti,ab | 72 |
| 27 | mydiagnostic*:ti,ab | 4 |
| 28 | microlife or WatchBP or "watch BP":ti,ab | 72 |
| 29 | heartscan or zenicor or aliveECG or kardia:ti,ab | 74 |
| 30 | photoplethysmograph or PPG:ti,ab | 1128 |
| 31 | (wearable) NEAR (technology or device* or sensor* or ECG or EKG or electrocardio*):ti,ab | 1726 |
| 32 | #1 or #2 or #3 | 10309 |
| 33 | #4 or #5 or #6 #7 or #8 or #9 or #10 or #11 or #12 or #13 or #14 or #15 or #16 or #17 or #18 or #19 or #20 or #21 or #22 or #23 or #24 or #25 or #26 or #27 or #28 or #29 or #30 or #31 | 54984 |
| 34 | #32 and #33 with Publication Year from 2023 to 2025, with Cochrane Library publication date Between May 2023 and Feb 2025, in Trials with 'Heart' in Cochrane Groups | 0 |

# **Section 4** – Web of Science Search Strategy

| **#** | **Searches** | **Results** |
| --- | --- | --- |
| 1 | TS="atrial fibrillation" | 140720 |
| 2 | TS=((atrial OR atria OR atrium OR auricular) NEAR/3 fibrillat*) | 142166 |
| 3 | TS=‘AF’ | 86957 |
| 4 | #1 OR #2 OR #3 "[atrial fibrillation]" | 142166 |
| 5 | TS="Mass screening" | 7365 |
| 6 | TS=((targeted OR opportunistic) NEAR/2 (detect* OR screen*)) | 82869 |
| 7 | TS="point of care" | 49542 |
| 8 | TS= "electronic health record" | 21990 |
| 9 | TS=((diagnos* NEAR/3 (performance* OR accurac* OR utilit* OR value* OR efficien* OR effectiveness))) | 249198 |
| 10 | TS=("electronic patient record*" OR "electronic medical record*" OR "electronic health record*" OR "computeri*ed patient record*" OR "computeri*ed medical record*" OR "computeri*ed health record*" OR EHR OR EPR) | 160887 |
| 11 | TS=((portable OR ambulatory OR monitor* OR lead* OR handheld OR "hand held" OR daily OR longterm OR short-term OR strap* OR device*) NEAR/3 (ECG* OR EKG* OR electrocardio*)) | 32821 |
| 12 | TS= ((ECG* OR EKG* OR electrocardio*) NEAR/2 (assess* OR check* OR monitor* OR detect* OR screen* OR diagnos* OR measure*)) | 2740 |
| 13 | TS=(((ambulatory OR event) NEAR/0 monitor*)) | 6744 |
| 14 | TS=(electrocardiography OR "ambulatory electrocardiography") | 26421 |
| 15 | TS=((ILR* OR "loop record*")) | 3673 |
| 16 | TS=(((heart OR cardiac) NEAR/0 monitor*)) | 5759 |
| 17 | TS=((pulse NEAR/2 (assess* OR check* OR monitor* OR detect* OR screen* OR diagnos* OR measure* OR palpation*))) | 45368 |
| 18 | TS=(("pulse oximetr*" NEAR/0 device*)) | 57 |
| 19 | TS=((("blood pressure" OR BP) NEAR/2 (assess* OR check* OR monitor* OR detect* OR screen* OR diagnos* OR measure*))) | 61904 |
| 20 | TS=("Blood Pressure Monitor" OR "Blood Pressure Monitoring") | 13255 |
| 21 | TS=((AliveCor OR MyDiagnostic*)) | 175 |
| 22 | TS=((Microlife OR WatchBP OR "watch BP")) | 177 |
| 23 | ALL=((Heartscan OR Zenicor OR AliveECG OR Kardia*)) | 1290 |
| 24 | ALL=((photoplethysmograph* OR PPG)) | 24278 |
| 25 | TS=((wearable NEAR/2 (technology OR device* OR sensor* OR ECG OR EKG OR electrocardio*))) | 57910 |
| 26 | TS=((smartwatch* OR "smart watch*" OR Applewatch* OR "Apple watch*" OR "wrist watch*" OR wristwatch* OR "fitness band*" OR "fitness tracker*" OR smartphone* OR "smart phone*" OR "mobile phone*")) | 143050 |
| 27 | TS=((wearable NEAR/2 (technology OR device* OR sensor* OR ECG OR EKG OR electrocardio*))) | 57910 |
| 28 | TS=("randomized controlled trial") | 229082 |
| 29 | TS=("randomized controlled trial") | 22082 |
| 30 | TS=(random*) | 270794 |
| 31 | TS=(factorial*) | 229082 |
| 32 | TS=((crossover* OR "cross over*")) | 178623 |
| 33 | TS=(((doubl* OR singl*) NEAR/0 blind*)) | 393681 |
| 34 | TS=((assign* OR allocat* OR volunteer* OR placebo*)) | 1829023 |
| 35 | TS=("crossover procedure") | 73 |
| 36 | TS=("single blind procedure") | 21 |
| 37 | TS=("double blind procedure") | 228 |
| 38 | #32 OR #33 OR #34 OR #35 OR #36 OR #37 [RCTs] | 325 |
| 39 | ALL=("Prospective studies") | 53077 |
| 40 | ALL=((prospective AND (study OR studies OR review OR analys* OR cohort* OR data))) | 829470 |
| 42 | TS=#38 OR #39 "[prospective studies]" | 882547 |
| 42 | TS=##38 OR #42  [RCTS OR "prospective studies]" | 882,872 |
| 43 | TS=#4 AND #31 AND #42 | 9284 |
| 44 | TS="case study" OR "case report" | 609,503 |
| 45 | TS=Editorial | 160,170 |
| 46 | TS=note | 965,452 |
| 47 | TS=letter OR letter | 4624 |
| 48 | TS=letter OR comment* | 770,093 |
| 49 | ALL=#44 OR #45OR #46 OR #47 OR #48 "[excluded publication types]" | 3764 |
| 51 | ALL=”Experiment" | 80 |
| 52 | ALL="Experimental Animal" | 3746 |
| 53 | TS="animal model" | 126,008 |
| 54 | TS=#51 OR #52 OR #53  "[animal studies]" | 189,786 |
| 55 | TS=#49 NOT #52 | 60,432 |
| 55 | TS=#55NOT #64[final results excluding animals"OR"certain excluded publication types]" | 46,244 |
| 56 | TS=#55 Timespan-1990-01-01 to 2025-02-01 | 15,567 |

# **Section 5:** Definitions

**Photoplethysmography (PPG):** non-invasive method for measuring blood volume changes in microvascular bed of skin based on optical properties such as absorption, scattering and transmission properties of human body composition under a specific light wavelength. ^1^

PPG based AF technology utilising PPG signal for opportunistic AF detection has been developed by multiple smartwatch manufacturers including Apple^2^, Samsung ^3^and Fitbit^4^.

| Types of Rhythm Monitoring | Description |
| --- | --- |
| Continuous ECG | ECG monitoring using a device worn throughout the study period, e.g. a patch |
| Intermittent ECG | Assessment with PPG when individual interacts with device at pre-determined time points in the study protocol e.g. four times per day with a handheld device |
| Continuous PPG | Continuous PPG assessment of underlying heart rhythm using a wearable device, e.g. watch |
| Intermittent PPG | Assessment with PPG when individual interacts with device at pre-determined time points in the study protocol e.g. four times per day |

**Systematic AF screening**: systematic screening programmes for AF differ from routine practice by offering tests for AF to a wider range of people than those who present in routine consultations with symptoms, risk factors or other indications for AF testing. A systematic approach would define which test should be used in conjunction with which screening strategy to increase the diagnosis of AF in the community among patients with asymptomatic AF and those who are symptomatic but remain undiagnosed.

**Study designs:**

We included randomised clinical trials of systematic AF screening that reported results of comparing an intervention arm (systematic AF screening) and control arm (usual care).

We included prospective studies where systematic AF screening had been conducted for individuals without a known AF diagnosis. These studies were not reported with a comparison arm of usual care. These studies were grouped under a title of prospective cohort studies.

**PICO:**

| Randomised clinical trials with an intervention and control arm |  |
| --- | --- |
| Patient, problem, or population | People aged ≥18 years |
| Intervention | Systematic screening for atrial fibrillation with a non-invasive device |
| Comparison, control, or comparator | People undergoing usual care |
| Outcome | 1. Detection of previously undiagnosed atrial fibrillation 2. Clinical outcomes including death, stroke, systemic embolization, cardiovascular hospitalisation, and bleeding |

| Prospective cohort studies |  |
| --- | --- |
| Patient, problem, or population | People aged ≥18 years without known diagnosis of atrial fibrillation |
| Exposure | Systematic screening for atrial fibrillation with a non-invasive device |
| Comparison, control, or comparator | - |
| Outcome | 1. Detection of previously undiagnosed atrial fibrillation |

**Risk of Bias assessment**

Cochrane Reviews provides framework for assessment of risk of bias in studies. When randomized controlled trials (RCT’s) are included, risk of bias is estimated using the revised version of Cochrane tool, RoB2. Prospective cohort studies were assessed through Cochrane’s ROBINS

RoB2 assessment is composed of the following domains:

1. Bias arising from the randomization process;
2. Bias due to deviations from intended interventions;
3. Bias due to missing outcome data;
4. Bias in measurement of the outcome; and
5. Bias in selection of the reported result.

Cochrane’s ROBIN assessment is composed of following domains:

1. Bias arising from confounding
2. Bias due to selection of participants
3. Bias in classification of interventions
4. Bias due to deviations from intended interventions
5. Bias due to missing outcome data;
6. Bias in measurement of the outcome
7. Bias in selection of the reported result

Each domain contains ‘signalling questions’, answers to which through aid of an algorithm, allow users to formulate judgement with respects to degree of bias for that particular domain. Potential answers to these signalling questions are as follows:

- Yes;
- Probably yes;
- Probably no;
- No;
- No information.

Once these questions have been answered, the subsequent steps involve concluding **risk to bias judgement** for that domain based on three levels:

- Low risk of bias;
- Some concerns; or
- High risk of bias.

**RCT’s** were overall scored low vs moderate vs high risk of bias as following:

**Low risk:** If all domains scored ‘low risk’ of bias

**Moderate risk**: If < 3 domains scored ‘some concerns’

**High risk**: If 1 or more domain scored ‘high risk’ of bias.

**Prospective cohort studies** were scored low/ moderate /high risk of bias as following:

**Low Risk:** If all domains scored ‘low risk’ of bias

**Moderate Risk**: If ≤ 4 domains scored ‘some concerns’

**High Risk**: If 1 or more domain scored ‘high risk’ of bias.

The scoring categories were agreed by co-authors prior to undertaking risk of bias assessment for each study.

# Figure 1: Forest plot of incidence rate of new atrial fibrillation diagnosis in prospective cohort studies or reports of the intervention arm alone from a randomised clinical trial of AF.


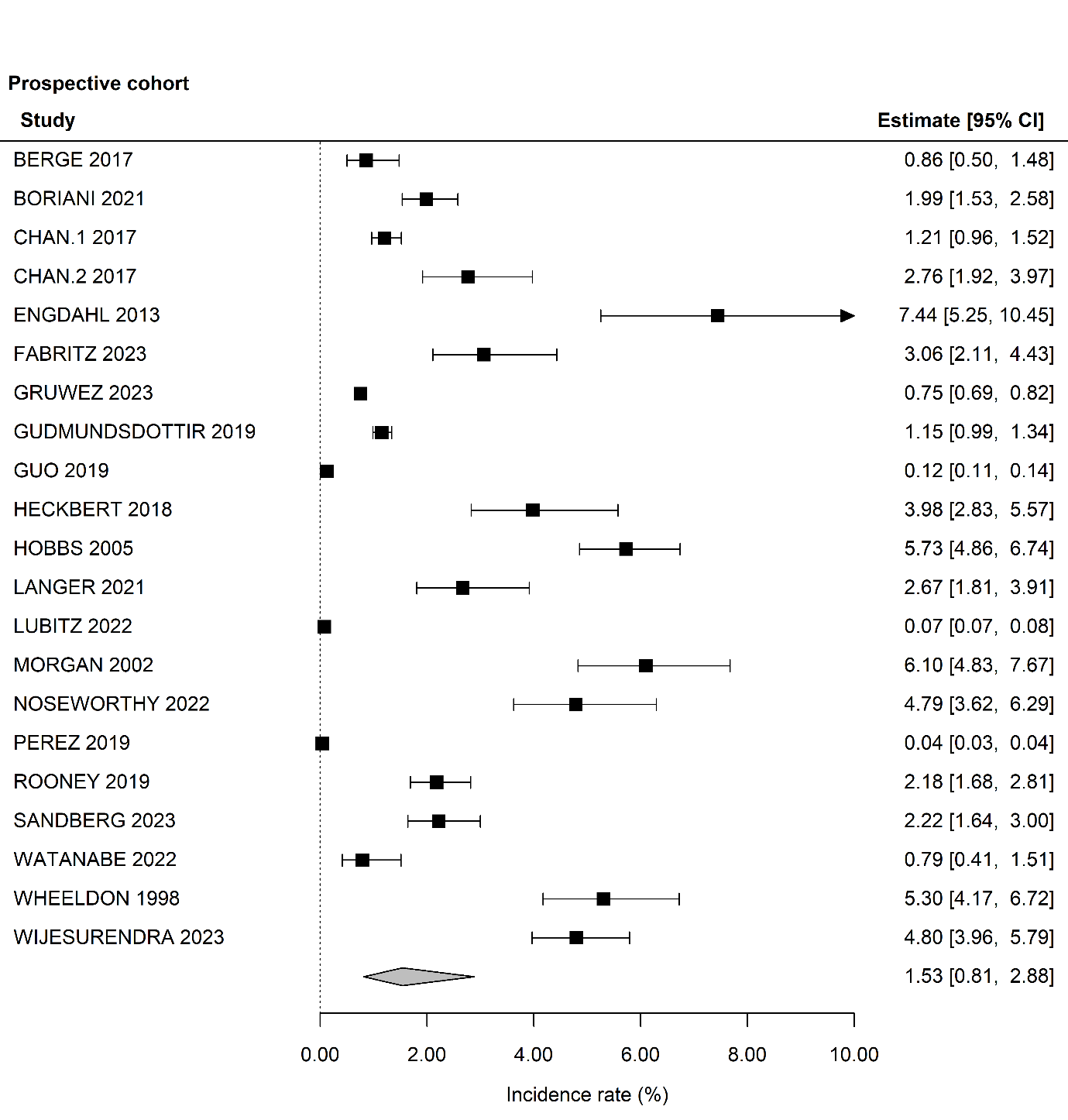


# Figure 2: Forest plot of incidence rate of new atrial fibrillation diagnosis in prospective cohort studies or reports of the intervention arm alone from a randomised clinical trial of AF, stratified by monitoring type.


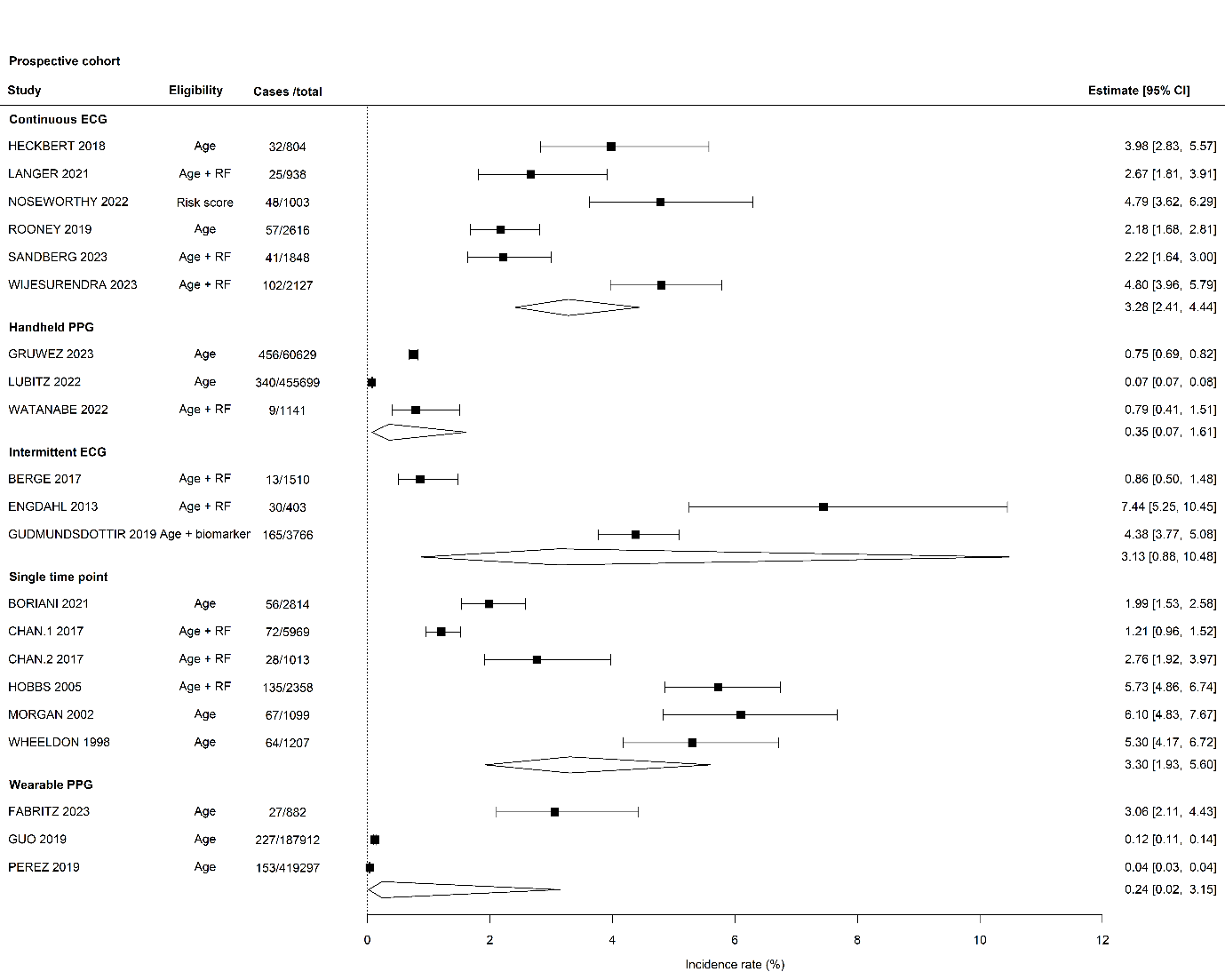


# Figure 3: Forest plot of incidence rate of new atrial fibrillation diagnosis in prospective cohort studies or reports of the intervention arm alone from a randomised clinical trial of AF, stratified by monitoring type limited to studies published after 2010.


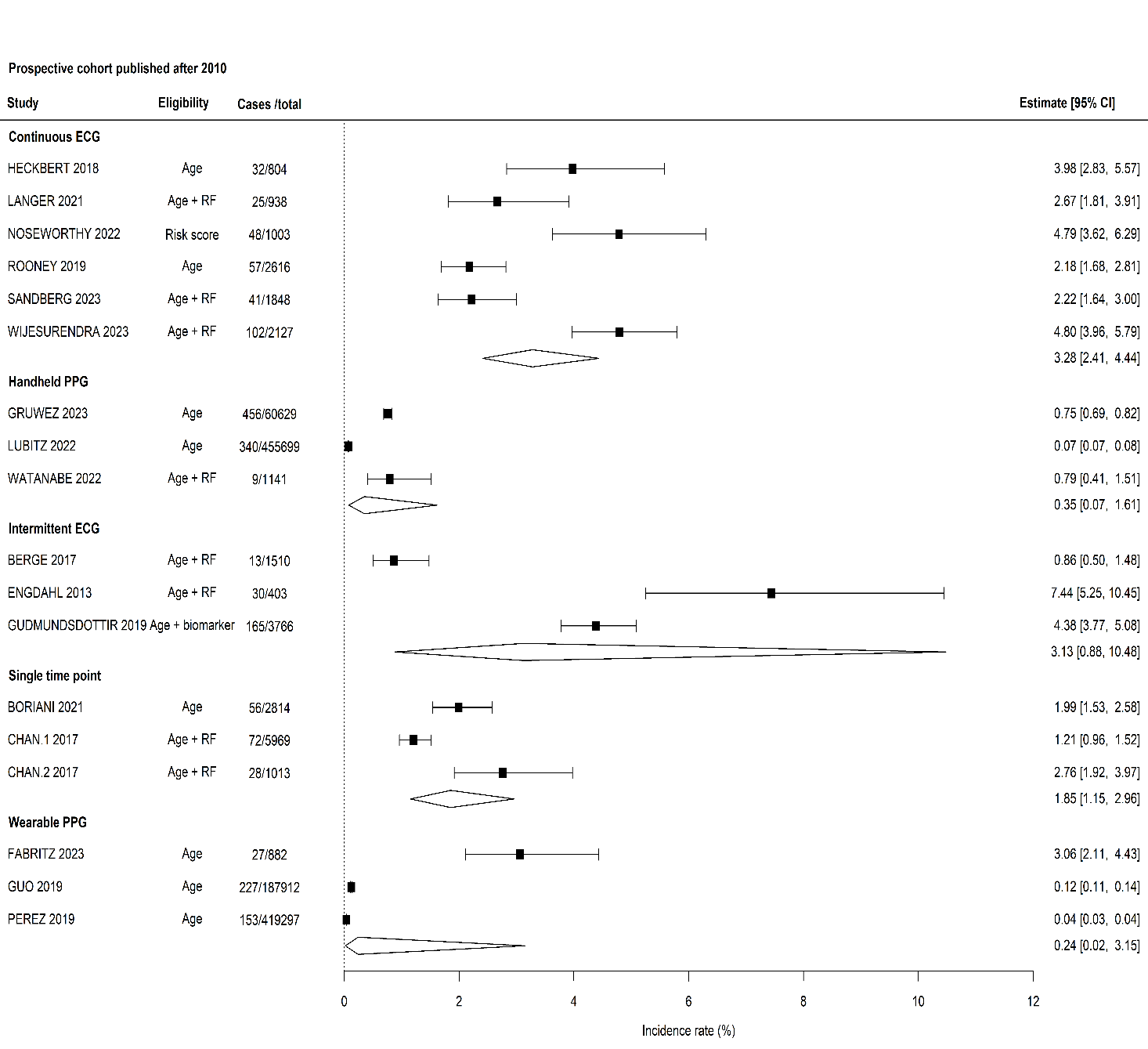


# **Figure 4:** Funnel plot for results of yield from prospective cohort studies


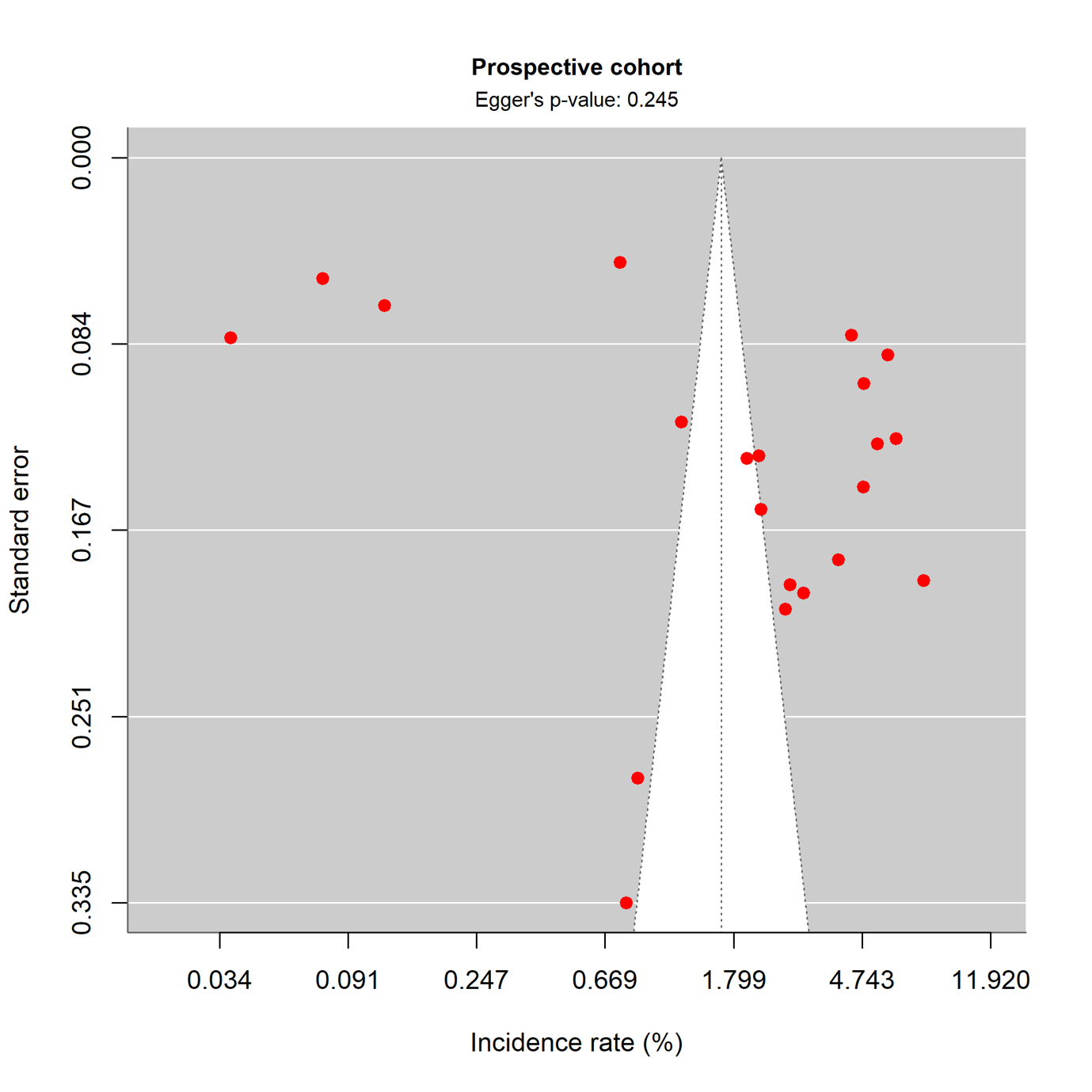


# **Figure 5**- Funnel plot of for results of clinical outcomes from randomised clinical trials


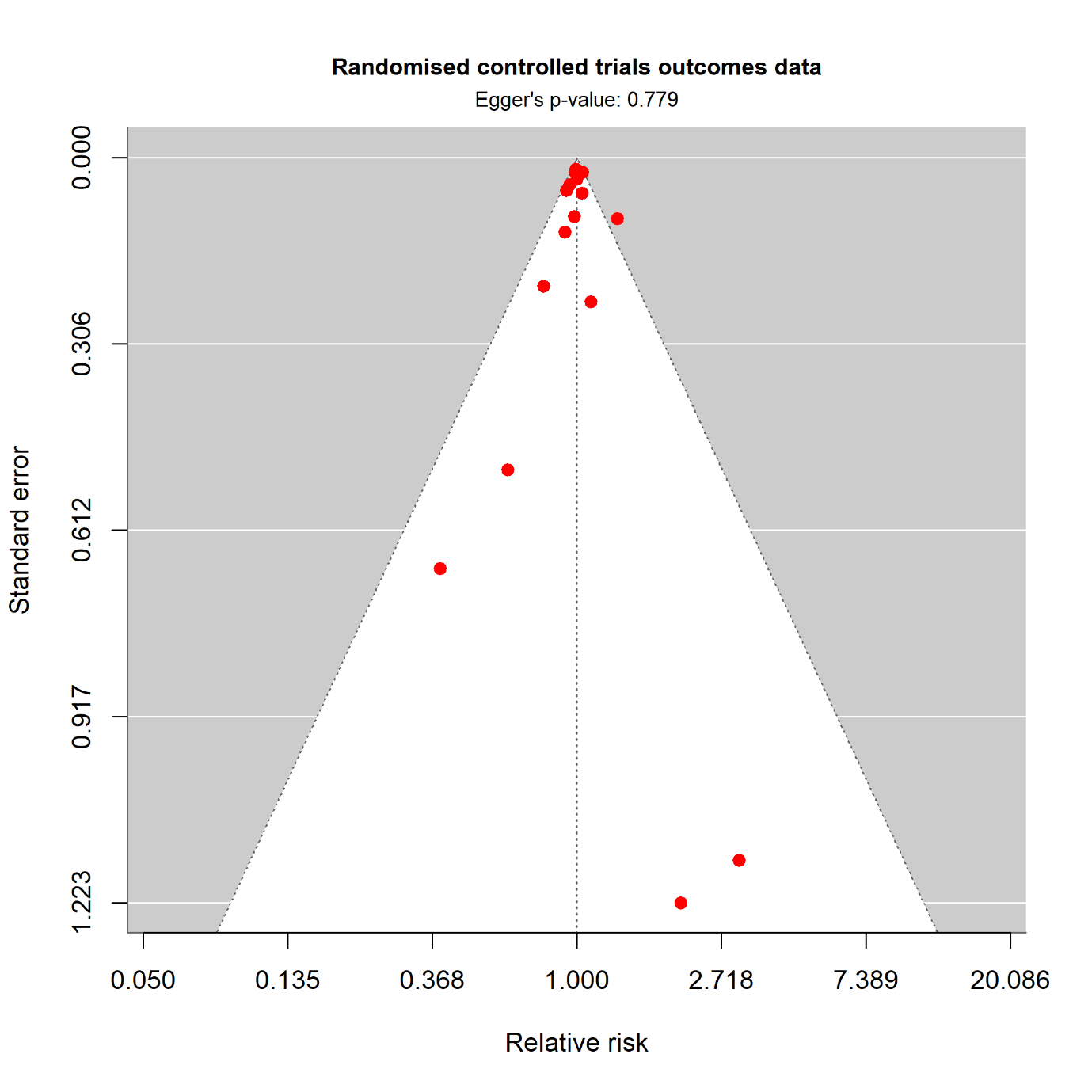


# **Figure 6**- Risk of Bias for prospective cohort studies.


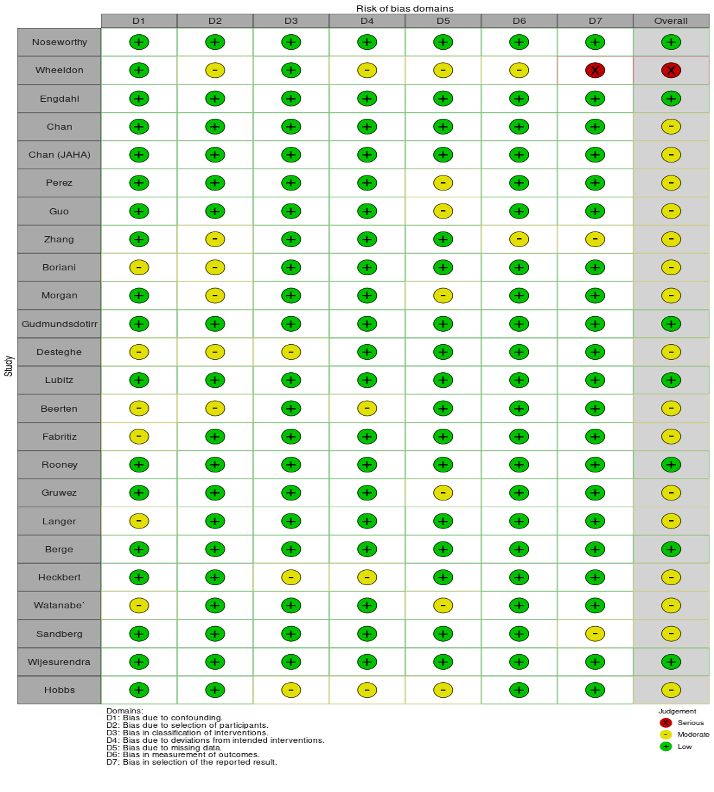


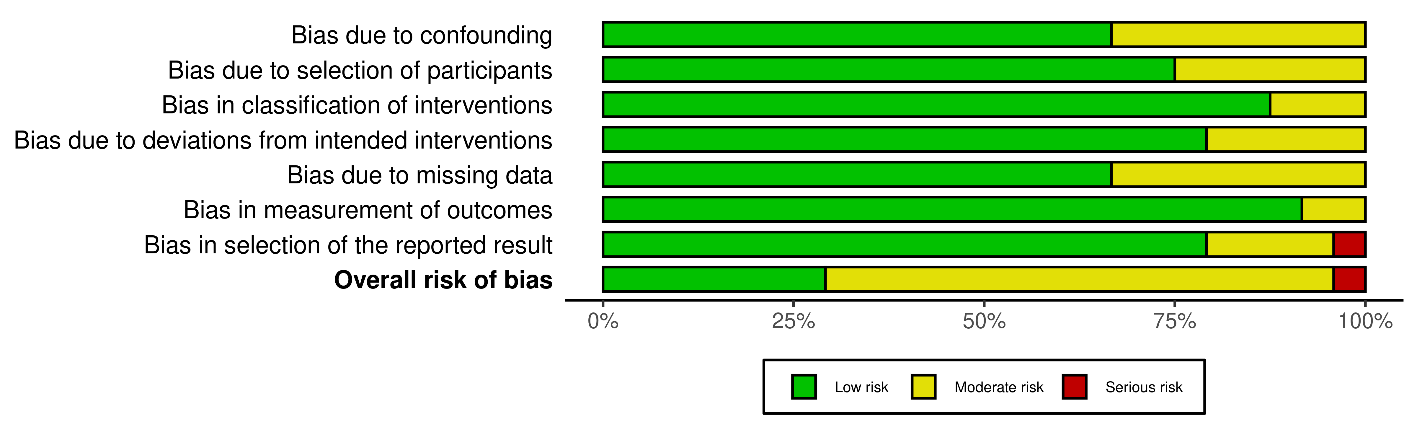


# **Figure 7**- Risk of Bias for RCT’s


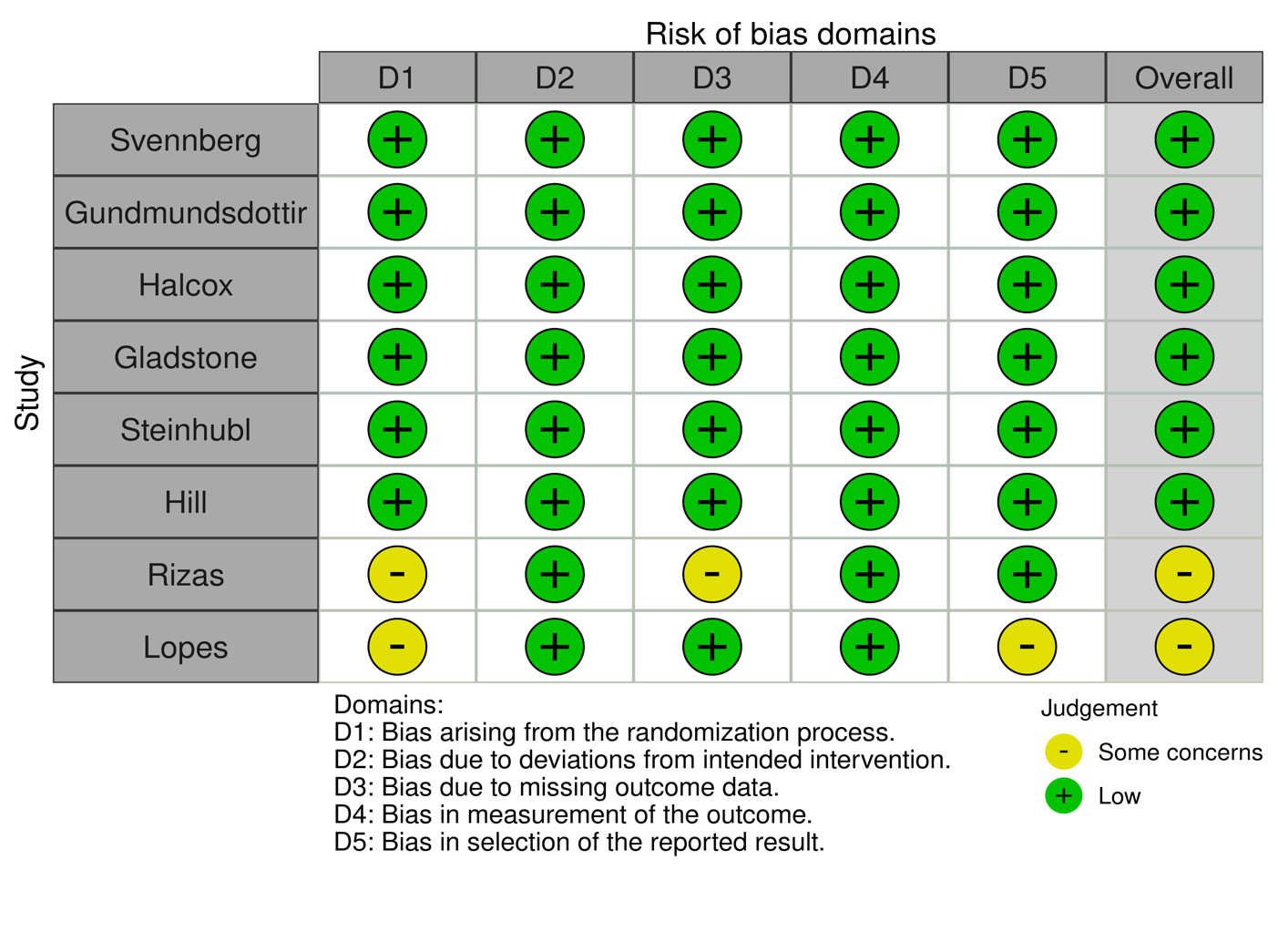


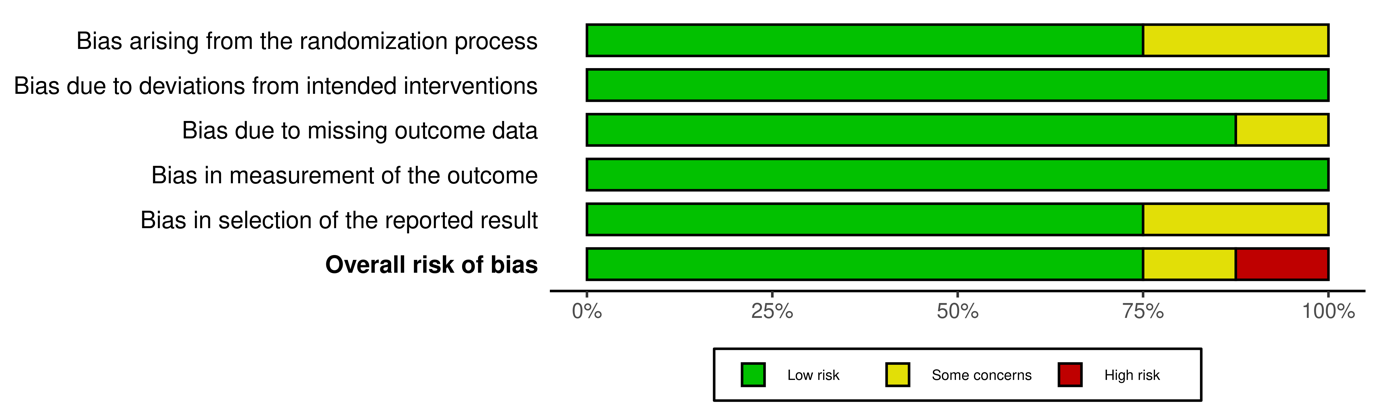


# **Figure 8:** Forrest plot after exclusion of high ROB prospective studies


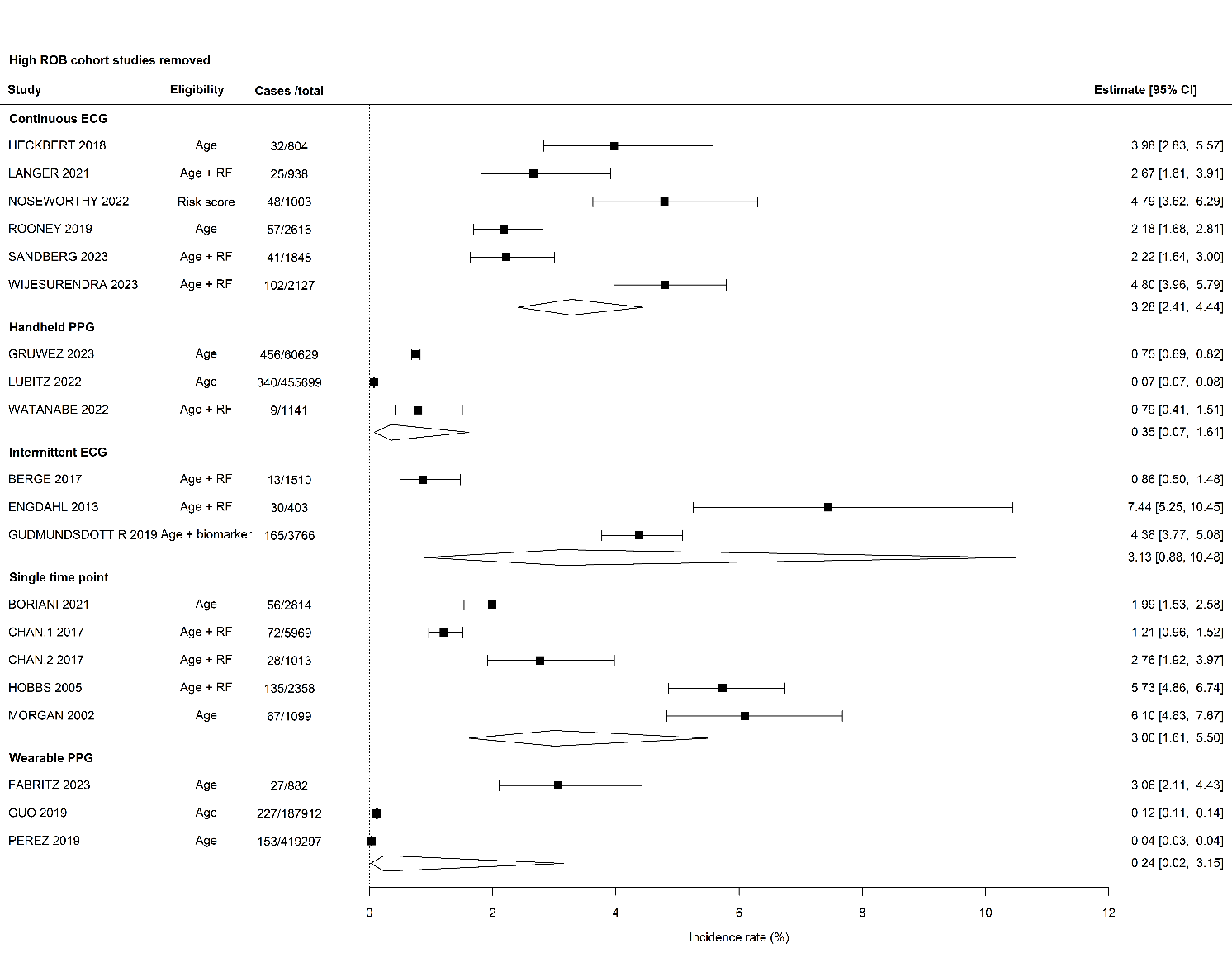


*Abbreviations: PPG- Photoplethysmography, ROB- Risk of Bias*

#

# **Table 1**- Baseline characteristics of RCT

| **Study (year)** | **Number** | **Location** | **Eligibility** | **Eligibility criteria** | **Exclusion criteria** | **Method of invitation** | **Method of consent** | **Method of device delivery** | **Protocol** | **Device** |
| --- | --- | --- | --- | --- | --- | --- | --- | --- | --- | --- |
| Svennberg et al  2021 | 28768 | Sweden | Age | 75-76 | No exclusion stated | Up to 3 written letters | Written | Attend screening centre | Single Lead ECG intermittently for 14 days | Zenicor |
| Gudmundsdottir et al  2019  (Sweden) | 28712 | Sweden | Age + Biomarker | 75-76+NT-proBNP≥125ng/L | No exclusion stated | Up to 2 written letters | Written | Attend screening centre | Single Lead ECG intermittently for 14 days | Zenicor |
| Halcox et al (2017) | 5846 | USA | >=65 + CHADSVASC>=2 | Age + 1 risk factor | <65, previous diagnosis of AF | Written letter | Written | Attend screening centre | twice-weekly single lead ECG for 12 months | AliveCor |
| Gladstone et al (2021) | 856 | Canada and Germany | Age >=75 with hypertension | Age + AF risk score (CHADVAS ≥2) | Previous diagnosis of AF/ Pacemaker insitu/ ILR | During outpatient appointment (Canada), written letters (Germany) | Written | Delivery | 2-week continuous ECG patch worn twice three weeks apart | ZioXT patch |
| Steinhubl et al (2018) | 2820 | USA | Male age > 55 years, or females age > 65 years + 1 comorbidity | Age + 1 risk factor | Previous diagnosis of AF/ Pacemaker insitu/ ILR/ already on NOAC | Email or written letter | Digital | Delivery | 4-week continuous ECG patch | ZioXT patch |
| Hill et al (2020) | 23745 | UK | >=30 | Risk Score | Previous diagnosis of AF | Up to 2 written letters and option of phone for non-responders | Written | Attend screening centre | twice-daily single-lead ECG for 2 weeks | AliveCor |
| Rizas et al (2022) | 2780 | Germany | 50-90, CHADSVASc ≥1 in men, CHADSVASc ≥2 in women, | Age + RF | Previous diagnosis of AF/ on NOAC | written letter | Digital | Delivery | PPG on smartphone app twice daily for 14 days then twice weekly until 6 months, if abnormal findings then 14-day ECG recorder | Smartphone Application |
| Singer et al (2024) | 11957 | USA | >=70 | Age | Previous diagnosis of AF/ on NOAC/pacemaker in situ | written letter | Written | Delivery | 14 days continuous ECG patch | ZioXT patch |

*Abbreviations: ILR- implantable loop recorder, NOAC- Novel Oral anticoagulation, PPG- Photoplethysmography*

*NT pro-BNP- Natriuretic pro Brain Natriuretic Peptide*

# **Table 2**- Baseline characteristics of prospective cohort studies.

| **Study (year)** | **N** | **Eligibility** | **Eligibility criteria** | **Exclusion criteria** | **Method of invitation** | **Method of consent** | **Method of device delivery** | **Protocol** | **Device** |
| --- | --- | --- | --- | --- | --- | --- | --- | --- | --- |
| Noseworthy et al  2021  (USA) | 1003 | >=18 | Risk score | Patient on NOAC/oral anticoagulation | Email/written letter | Digital | Delivery | 30-day continuous ECG patch | MoMe 3-lead monitor |
| Hobbs et al  2005  (UK) | 2358 | >65 + Risk factor | Age + Risk factor | Palliative/ change in registered general practice | Up to two written letters | Written | Attended screening clinic | 12- Lead ECG screening clinic | 12 lead ECG |
| Morgan  2002  (UK) | 1099 | >=65 | Age | No exclusion criteria stated | Up to 2 written letters and option of phone for non-responders | Written | Attended screening clinic | Pulse palpation followed by ECG | 12 lead ECG |
| Wheeldon et al  1998  (UK) | 1207 | ≥65 years | Age | No exclusion criteria stated | Up to 2 written letters and option of phone or visit for non-responders | Written | Attend screening centre | 12- Lead ECG screening clinic | 12- Lead ECG |
| Engdahl et al  2013  (Sweden) | 848 | 75-76 | Age + Risk factor | <75 years | Up to two written letters | Written | Attend screening centre | Twice-daily single-lead ECG for 2 weeks | Zenicor II handheld device |
| Chan et al  2017  (Hong Kong) | 5969 | Age ≥ 65  OR  diabetes mellitus /hypertension | Age or Risk factor | Patients with pacemaker in situ | In person in outpatient clinic | Written | In person in outpatient clinic | Single lead ECG | AliveCor Heart monitor |
| Chan et al  2017  (Hong Kong) | 1013 | Age ≥65  OR  diabetes mellitus /hypertension | Age  OR  Risk factor | Patients with pacemaker or ICD in situ | In person in outpatient clinic | Written | In person in outpatient clinic | Single lead ECG | AliveCor KardiaMobile Heart Monitor |
| Perez et al  2019  (USA) | 419297 | Age ≥ 22  With  Apple iPhone+ watch + ECG patch notification | Age | Previous diagnosis of AF | Available App | Digital | Consumer-owned device | PPG on smartphone followed by ECG confirmation | Apple watch photoplethysmography sensor |
| Guo et al  2019  (China) | 187912 | Age ≥ 18  With  Huawei (android 5.0) + watch | Age | <18 and inability to use smartphone | Available app | Digital | Consumer-owned device | PPG on smartphone followed by ECG confirmation | Wristwatch (Huawei watch GT/Honor watch) /Wristband (Honor band 4) |
| Lubitz et al  2022  (USA) | 455699 | Age ≥ 22 | Age | Previous diagnosis of AF, on NOAC,  PPM/ICD in situ | Email,  FitBit app notifications,  Social media,  Other marketing channels | Digital | Consumer-owned device | PPG data Fitbit followed by ECG confirmation | Fitbit device paired with FitBit account on android/IOS smartphone |
| Boriani et al  2021  (Italy) | 2814 | Age ≥ 18 | Age | Previous diagnosis of AF,  PPM/ICD in situ | Not stated | Written | Delivery | Single lead ECG followed by 12 lead ECG | MyDiagnostick bar device |
| Beerten et al  2021  (Belgium) | 92 | Age ≥ 65,  AF risk score (CHARGE AF of at least 10%) | Risk score | Previous diagnosis of AF,  PPM/ICD in situ | Not stated | Written | Consumer-owned device | Twice daily use of Fibricheck app for 14 days | Fibricheck app |
| Fabritz et al  2023  (Belgium/Germany/Poland) | 882 | Age ≥ 65 | Age | Previous diagnosis of AF,  On oral anticoagulants | Newspaper and TV advertisements targeting older adults,  Senior citizen interest groups,  Personal contacts in the sites,  GP in the community,  Leaflets and a website | Digital | Delivery | PPG wristband,  Followed by 14-day ECG recorder in the event of irregular pulse | Corsano Preventicus app |
| Rooney et al  2019  (USA) | 2616 | Age 45-64 at inception of cohort | Age | ILR/PPM/ICD in situ,  On oral anticoagulants | In prospective cohort study | Not applicable | In prospective cohort study | Continuous ECG for 14 days | ZioXT patch |
| Gruwez et al  2023  (Belgium) | 60629 | Age ≥ 65 | Age | No clear exclusion criteria | Article in newspaper | Digital | Consumer-owned device | PPG via smartphone app,  Followed by questionnaire,  Confirmation via 12 lead ECG | Fibricheck app |
| Langer et al  2021  (Canada) | 938 | Age ≥ 65 with ≥ 1 high-risk factor  OR  Age ≥ 75 with ≥ 2 moderate risk factors | Age  And  Risk factor | Previous diagnosis of AF on 12 lead ECG/Holter,  PPM/ICD in situ | In person in outpatient clinic | Written | In person in outpatient clinic | Continuous ECG for 7 days | CardioState  Icentia, Canada |
| Heckbert et al  2018  (USA) | 804 | Age 45- 84 at inception of cohort  Without clinically recognized cardiovascular disease | Age | Allergy to tapes and adhesives, | In prospective cohort study | Not applicable | In prospective cohort study | ≥ 12 days continuous monitoring with one device  VS.  ≥ 2 days continous monitoring with two devices | ZioXT patch |
| Berge et al  2017  (Norway) | 1510 | Age ≥ 65,  CHA2DS2-VASc score  ≥ 2 (men)  ≥ 3 (women) | Age + RF | Known AF | In prospective cohort study | Not applicable | In prospective cohort study | Twice daily single- lead ECG for 2 weeks | Zenicor II handheld device |
| Watanbe et al  2022  (Japan) | 1141 | Age ≥ 65,  With  Moderate-to-high risk of stroke  CHA2DS2-VASc score  ≥ 2  Or  CHADS2 > 1 | Age + RF | Previously documented AF,  Use of anti-arrhythmic drugs,  Inability to use monitoring devices properly | In person in outpatient clinic | Written | In person in outpatient clinic | Patient undergoing BP monitoring,  Followed by  Twice daily single-lead ECG via handheld device for 14 days | HEM-9200T blood pressure cuff, then  MyBeat Omron handheld device |
| Zhang et al  2022  (China) | 3244 | Age ≥ 65 | Age | Previous diagnosis of AF | Public health press conference and media release in Shanghai  Official notices of the neighbourhood committee,  Posters in 5 community health centers | Written | Attend screening center | Screening every year with single-lead ECG | AliveCor KardiaMobile handheld device |
| Zhang et al  2022  (China) | 2944 | Age ≥ 65 | Age | Previous diagnosis of AF | Public health press conference and media release in Shanghai  Official notices of the neighbourhood committee,  Posters in 5 community health centers | Written | Attend screening center | Screening every quarter-year with single-lead ECG | AliveCor KardiaMobile handheld device |
| Sandberg et al  2023  (Norway) | 1848 | Age ≥ 65  with  > 1 risk factor for stroke according to CHA2DS2-VASc risk score | Age + RF | Self-reported prior diagnosis of AF,  No access to a smart phone | Hospitals Facebook pages,  Study sponsor’s (pharmaceutical companies) Facebook pages,  Mention in regional newspapers and on radio | Digital | Delivery | Up to 14 days continuous ECG patch | ECG 247 Smart Heart sensor |
| Wijesurendra et al  2023  (UK) | 2127 | Age ≥ 65  With  CHA2DS2-VASc score  ≥ 3 (men)  ≥ 4 (women)  With  No previous history of AF  And  No latex allergy | Age + RF | Previous diagnosis of AF | Written letter | Written | Delivery | 14 days continuous ECG patch | ZioXT patch |

*Abbreviations: N- number of participants, PPM- Permanent Pacemaker*

# **Table 3**: Characteristics of recruitment and protocol adherence (RCT’s)

| **Study (Country)** | **Number invited overall** | **Number consented overall (%)** | **Number invited intervention** | **Number invited control** | **Number consented intervention** | **Number consented control** | **PPG conducted** | **PPG irregular notification** | **ECG rhythm monitoring conducted** | **AF in control arm (%)** |
| --- | --- | --- | --- | --- | --- | --- | --- | --- | --- | --- |
| Svennberg 2021 (Sweden) | 28768 | N/A | 14387 | 14381 | 14387 | 14381 | No | N/A | 7173 | 3 |
| Gudmundsdottir et al  2019  (Sweden) | 28712 | N/A | 13870 | 14356 | 3766 | 2549 | No | N/A | 3766 | 0.04 |
| Halcox et al  2017  (UK) | 5846 | 17.1 | 502 | 502 | 502 | 502 | No | N/A | 500 | 3.8 |
| Gladstone et al  2021  (Canada/Germany) | 856 | 100 | 434 | 422 | 434 | 422 | No | N/A | 386 | 5.3 |
| Steinhubl et al 2018  (USA) | 2820 | 94.2 | 1366 | 1293 | 1366 | 1293 | No | N/A | 906 | 3.9 |
| Hill et al  2020  (UK) | 23745 | 3.3 | 11849 | 11896 | 11849 | 11896 | No | N/A | 255 | 5.1 |
| Rizas et al  2022  (Germany) | 5551 | 100 | 2860 | 2691 | 2860 | 2691 | No | N/A | 2142 | 4 |
| Singer et al  2024  (USA) | 11957 | 11905 | 11957 | 5953 | 5952 | 5332 | No | N/A | 5684 | 3.3 |

*Abbreviations PPG- Photoplethysmography*

# **Table 4:** Characteristics of recruitment and protocol adherence (observational studies)

| **Study (Country)** | **Number invited overall** | **Number consented overall (%)** | **Number consented intervention**  **n** | **PPG conducted** | **PPG irregular notification** | **ECG rhythm monitoring conducted** | **AF in intervention arm (%)** |
| --- | --- | --- | --- | --- | --- | --- | --- |
| Noseworthy  2021  (USA) | 15165 | 8.0 | 1225 | No | N/A | 1003 | 7.6 |
| Hobbs  2005  (UK) | 14802 | 29.9 | 4433 | No | N/A | 2357 | 5.7 |
| Morgan  2002  (UK) | 3001 | 49.9 | 1499 | No | N/A | 1099 | 4.5 |
| Wheeldon et al  1998  (UK | 1422 | 100 | 1422 | No | N/A | 1207 | 5.3 |
| Engdahl et al  2013  (Sweden) | 1330 | 63.7 | 848 | No | N/A | 403 | 7.2 |
| Chan et al  2017  (Hong Kong)  BMJ Open | 6075 | 98.2 | 5969 | No | N/A | 5969 | 1.2 |
| Chan et al  2017  (Hong Kong) | 1097 | 93.5 | 1026 | No | N/A | 1013 | 2.7 |
| Perez et al  2019  (USA) | 419297 | 100 | 419297 | 2161 | 2161 | 450 | 3.6 |
| Guo et al  2019  (China) | 187912 | 100 | 246541 | 265139 | 424 | 187912 | 0.1 |
| Lubitz et al  2022  (USA) | 455699 | 100 | 455699 | 4728 | N/A | 2513 | 15.3 |
| Boriani et al  2021  (Italy) | 2814 | 100 | 2814 | No | N/A | 1848 | 2 |
| Beerten et al  2021  (Belgium) | 92 | 100 | 92 | No | N/A | 92 | 5.7 |
| Fabritz et al  2023  (Belgium/Germany/Poland) | 1037 | 90.1 | 935 | N/A | 53 | 882 | 3.1 |
| Rooney et al  2019  (USA) | 15792 | 25.4 | 4003 | No | N/A | 2616 | 2.2 |
| Gruwez et al  2023  (Belgium) | 62807 | 100 | 62807 | 513821 | N/A | 60629 | N/A |
| Langer et al  2021  (Canada) | 942 | 100 | 942 | No | N/A | 942 | 2.7 |
| Heckbert et al  2018  (USA) | 1500 | 92 | 1385 | No | N/A | 1385 | N/A |
| Berge et al  2017  (Norway) | 3706 | 43.2 | 1601 | No | N/A | 1510 | 0.9 |
| Watanbe et al  2022  (Japan) | 1148 | 99 | 1141 | N/A | 481 | 480 | 0.09 |
| Zhang et al  2022  (China) | 8420 | 100 | 8240 | No | N/A | 576 | N/A |
| Zhang et al  2022  (China) | 8420 | 50 | 4120 | No | N/A | N/A | N/A |
| Sandberg  2023  (Norway) | N/A | N/A | 2212 | No | N/A | 1849 | 41 |
| Wijesurendra  2023  (UK) | 22188 | 22.7 | 5040 | No | N/A | N/A | 4.8 |
| Singer et Al  2024  (USA) | 11957 | 49.8 | 5952 | No | No | 5684 | 5.0 |

*Abbreviations PPG- Photoplethysmograhy*

# Table 5: Baseline characteristics of all studies

| **Study (Country)** | **N** | **Age (mean, SD)** | **Female %** | **Hypertension %** | **Diabetes mellitus %** | **Heart failure %** | **Stroke/TIA %** | **CHA2DS2-VASC score (mean, SD)** |
| --- | --- | --- | --- | --- | --- | --- | --- | --- |
| Svennberg et al  2021  (Sweden) | 28768 | 75.5 (-) | 53.9 | 31.6 | 11.6 | 4.8 | 8.8 | 3.3; (-) |
| Halcox et al  2017  (UK) | 5846 | 72.6 (5.4) | 53.4 | 53.9 | 26 | 1 | 7 | 3; 1 |
| Gladstone et al  2021  (Canada/Germany) | 856 | 79.8 (3.8) | 56.9 | N/A | 23.9 | 4.1 | 9.7 | 4; (-) |
| Steinbuhl et al  2018  (USA) | 2820 | 73.5 (7.4) | 36.3 | 72.6 | 35.5 | 4.5 | 13.1 | 3; (-) |
| Hill et al  2020  (UK) | 23745 | 78.4 (8.9) | 45.3 | 69.4 | 23.7 | 7.8 | 8.1 | N/A; (-) |
| Rizas et al  2022  (Germany) | 2780 | N/A (5.5) | 31 | 65 | 13 | 4 | 6 | 3; (-) |
| Noseworthy et al  2021  (USA) | 1003 | 75.2 (7.6) | 38.2 | 82.4 | 30.6 | 17.5 | 13.3 | 3.6; (-) |
| Hobbs  2005  (UK) | 2358 | 75.2 (7.1) | N/A | N/A | N/A | N/A | N/A | N/A; (-) |
| Morgan  2002  (UK) | 1099 | 75.3 (-) | 58 | N/A | N/A | N/A | N/A | N/A; (-) |
| Gudmundsdottir et al  2019  (Sweden) | 6315 | 75.5 (-) | 54 | 51.7 | 11.4 | 2.4 | 8.1 | 3.4; 1 |
| Wheeldon et al  1998  (UK | 1207 | (N/A) | (N/A) | 17.9 | N/A | 32.1 | 7.1 | N/A; (-) |
| Engdahl et al  2013  (Sweden) | 848 | 75.5 (-) | 57 | 53 | 11 | 4 | 10 | 1.9; (-) |
| Chan et al  2017  (Hong Kong) | 5969 | 67.2 (11) | 53.7 | 82.9 | 45.9 | 0.9 | 4.5 | 2.8; 1.3 |
| Chan et al  2017  (Hong Kong) | 1013 | 68.4 (12.2) | 53.2 | 90.4 | 36.6 | 4.4 | 10.5 | 3.0; 1.5 |
| Perez et al  2019  (USA) | 419297 | 41 (13) | 42 | 21 | 4.9 | 0.6 | 1 | 1; (-) |
| Guo et al  2019  (China) | 187912 | 34.7 (11.5) | 13 | N/A | N/A | N/A | N/A | 1; (-) |
| Lubitz et al  2022  (USA) | 455699 | (N/A) | 48.2 | 42.1 | 8.3 | 0.7 | 0.5 | 1.5; 1.1 |
| Boriani et al  2021  (Italy) | 2814 | (N/A) | 57.9 | 75 | 19.6 | 26.8 | 8.9 | 3.3;1 |
| Beerten et al  2021  (Belgium) | 92 | 78 (8.1) | 39 | 84 | 36.3 | 6.8 | 11.3 | N/A; (-) |
| Fabritz et al  2023  (Belgium/Germany/Poland) | 882 | 71 (5) | 65.7 | 47 | 11 | N/A | N/A | 2.6; 1.4 |
| Rooney et al  2019  (USA) | 2616 | 79.2 (4.6) | 57.9 | N/A | 32 | 8.2 | 4.1 | 3.8; 1.1 |
| Gruwez et al  2023  (Belgium) | 60629 | 49 (15) | 43 | 20 | 4 | 3 | 3 | 1; (-) |
| Langer et al  2021  (Canada) | 938 | 78.7 (6.1) | 42.1 | 64.1 | 30.5 | 10.4 | 69.2 | N/A; (-) |
| Heckbert et al  2018  (USA) | 804 | 75(8) | 49.1 | 54.3 | 14.8 | 3.8 | N/A | N/A; (-) |
| Berge et al  2017  (Norway) | 1510 | 65 (-) | 43.6 | 86.4 | 21.3 | 2.4 | 7.4 | 2.7; 0.8 |
| Watanbe et al  2022  (Japan) | 1141 | (N/A) | 49.5 | 92.5 | 29.1 | 5.4 | 2.2 | 4; (-) |
| Zhang et al  2022  (China) | 3244 | 71.3 (6.1) | 49.6 | 68.7 | 25.5 | 0.6 | 22.6 | N/A; (-) |
| Zhang et al  2022  (China) | 2944 | 71.6 (6.3) | (N/A) | N/A | N/A | N/A | N/A | N/A; (-) |
| Sandberg  2023  (Norway) | 1848 | 70.1 (4.2) | 73 | 47 | 8 | 2 | 3 | 2.6; 0.8 |

*Abbreviations: SD- Standard Deviation*

# **Table 6**- Cochran's Q, degrees of freedom and I^2 measures of statistical heterogeneity within subgroup analyses

| **Eligibility criteria** | Cohort studies | Randomised controlled trials |
| --- | --- | --- |
| **Age + RF** | Q = 204.36, df = 8, p = 0; I^2 = 96.6% | Q = 5.80, df = 3, p = 0.012; I^2 = 45.6% |
| **Age** | Q = 5229.8, df = 10, p = 0; I^2 = 99.8% | Q = 8.62, df = 1, p = 0.13; I^2 = 56.2% |
|  |  |  |
| **Monitoring type** |  |  |
| **Intermittent ECG** | Q = 44.27, df = 2, p = 0; I^2 = 97.7% | Q = 8.62, df = 2, p = 0.01; I^2 = 83.4% |
| **Continuous ECG** | Q = 39.87, df = 5, p = 0; I^2 = 85.9% | Q = 16.55, df = 2, p = 0; I^2 = 89.0% |
| **Single time point** | Q = 170.58, df = 5, p = 0; I^2 = 96.7% | - |
| **Intermittent PPG** | Q = 1051.77, df = 2, p = 0; I^2 = 99.7% | - |
| **Continuous PPG** | Q = 474.46, df = 2, p = 0; I^2 = 99.8%) | - |
|  |  |  |
| **Age criteria** |  |  |
| **75 years and older** | Q = 7.47, df = 1, p = 0.01; I^2 = 86.6% | Q = 12.52, df = 2, p = 0.07; I^2 = 85.7% |
| **65 - 74 years** | Q = 855.02, df = 11, p = 0; I^2 = 97.8% | Q = 3.4, df = 1, p = 0.07; I^2 = 70.6% |
| **45 - 64 years** | Q = 7.64, df = 1, p = 0.01; I^2 = 86.9% | - |
| **18 - 44 years** | Q = 1369.48, df = 4, p = 0; I^2 = 99.9% | - |

*Abbreviations : RF- Risk Factor, Q- Cochrane Q, 1^2- Heterogeneity statistic*
